# Systematic review and meta-analysis of the association between childhood overweight and obesity and primary school diet and physical activity policies

**DOI:** 10.1186/1479-5868-10-101

**Published:** 2013-08-22

**Authors:** Andrew James Williams, William E Henley, Craig Anthony Williams, Alison Jane Hurst, Stuart Logan, Katrina Mary Wyatt

**Affiliations:** 1 Institute of Health Services Research, University of Exeter Medical School (formerly Peninsula College of Medicine and Dentistry), Veysey Building, Salmon Pool Lane, EX2 4SG, Exeter, Devon, UK; 2 Children’s Health and Exercise Research Centre, Sport and Health Sciences, College of Life and Environmental Sciences, University of Exeter, St. Luke’s Campus, Heavitree Road, EX1 2LU, Exeter, Devon, UK

**Keywords:** Policy, Obese, Nutrition, Physical education

## Abstract

Obesity is a major public health concern and there are increasing calls for policy intervention. As obesity and the related health conditions develop during childhood, schools are being seen as important locations for obesity prevention, including multifaceted interventions incorporating policy elements. The objective of this systematic review was to evaluate the effects of policies related to diet and physical activity in schools, either alone, or as part of an intervention programme on the weight status of children aged 4 to 11 years. A comprehensive and systematic search of medical, education, exercise science, and social science databases identified 21 studies which met the inclusion criteria. There were no date, location or language restrictions. The identified studies evaluated a range of either, or both, diet and physical activity related policies, or intervention programmes including such policies, using a variety of observational and experimental designs. The policies were clustered into those which sought to affect diet, those which sought to affect physical activity and those which sought to affect both diet and physical activity to undertake random effects meta-analysis. Within the diet cluster, studies of the United States of America National School Lunch and School Breakfast Programs were analysed separately; however there was significant heterogeneity in the pooled results. The pooled effects of the physical activity, and other diet related policies on BMI-SDS were non-significant. The multifaceted interventions tended to include policy elements related to both diet and physical activity (combined cluster), and although these interventions were too varied to pool their results, significant reductions in weight-related outcomes were demonstrated. The evidence from this review suggests that, when implemented alone, school diet and physical activity related policies appear insufficient to prevent or treat overweight or obesity in children, however, they do appear to have an effect when developed and implemented as part of a more extensive intervention programme. Additional evidence is required before recommendations regarding the focus of policies can be made and therefore, increased effort should be made to evaluate the effect of policies and policy containing intervention programmes upon weight status.

## Introduction

Obesity among children is associated with significant psychological, social and health consequences including insulin resistance, cardiovascular disease, low self-esteem and poorer education and employment outcomes [1,2]. The rising prevalence of obese children combined with the increased likelihood of obesity continuing into adulthood, has resulted in childhood being seen as an important period for interventions to prevent overweight and obesity [3-5]. There are increasing calls for governments to implement policies which could halt the rise in obesity, similar to the policies initiated to address smoking [6,7]. Within the United States of American (USA) and the United Kingdom (UK) there have been guidelines and policies introduced by the governments to promote healthy behaviour in school children, however, the impact of such guidelines and policies is rarely evaluated scientifically [8-10]. This systematic review was conducted to examine the effect of school diet and physical activity related policies on anthropometric outcomes during primary education (primary or junior school in the UK, elementary school in the USA).

To date the systematic reviews which have examined the effect of obesity related school policies have evaluated diet and physical activity outcomes rather than weight status [11-16]. Jaime and Lock [11] and Van Cauwenberghe [14] identified policy components which appear to have a positive effect upon diet, including: nutrition guidelines; healthy food price interventions and fruit and vegetable distribution or subscription schemes. They found a lack of evidence for policies affecting children’s breakfast or unhealthy food choices [11,14]. Lagarde and LeBlanc [15] identified a number of studies which reported an increase in physical activity as a result of policy such as: improving the quality and variety of physical education (PE); mandatory qualifications for PE teachers and adequate facilities. This paper extends existing work by systematically reviewing the evidence for the effect of diet and physical activity policies on children’s weight status [11,14-16].

## Review

### Methods

Guidance from The Cochrane Collaboration and the National Health Service Centre for Reviews and Dissemination informed the development of the review protocol, which is available upon request [17,18].

#### Search strategy

Two search strategies were developed for this systematic review, one for diet related and one for physical activity related policies (Additional file 1). Each search strategy contained population terms, intervention terms and outcome terms with only the intervention terms differing between the two searches. Each set of terms included thesaurus terms or Medical Subject Headings (MeSH) as well as title and abstract text searches.

The following databases were searched from their earliest record to June 2011: Medline In-Process & Other Non-Indexed Citations [Ovid], Medline [Ovid], EMBASE [Ovid], PsychINFO [Ovid], SportDISCUS [Ebscohost], Web of Science [ISI Web of Knowledge], Education Resource Information Center (ERIC) [Dialog Datastar], British Education Index [Dialog Datastar], Australian Education Index [Dialog Datastar], Cumulative Index to Nursing and Allied Health Library (CINAHL Plus) [Ebscohost], and The Cochrane Library [Wiley Online]. The search strategy was developed in Medline (Additional file 1) prior to adaptation for the other databases (the complete search log is available upon request).

A grey literature search for unpublished and continuing research was undertaken in July 2011 in the metaRegister of Controlled Trials, Clinical Trials.gov and the International Clinical Trials Registry Platform [18]. Similarly the Robert Wood Johnson Foundation website was searched for items not published within journals [19]. The following search term was used ‘school and (physical activity or physical education or nutrition or diet) and policy’ with the age limiter ‘child’ where it was available. The references of included studies and systematic reviews were inspected for any additional studies.

#### Eligibility criteria

The eligibility criteria are outlined in Table 1. There was no date, geographic or language restrictions. The population of interest in this systematic review was children aged 4 to 11 years participating in full time education. The definition of policy utilised to identify whether an intervention was eligible was that defined by Milio [20].

‘Policy is a guide to action to change what would otherwise occur… Policy sets priorities and guides resource allocation.’

p622, Milio [20]

**Table 1:** Eligibility criteria

**Inclusion criteria**	**Exclusion criteria**
*Population:* children undertaking primary education aged between 4 and 11 years	*Population:* people outside the specified age range and animal models
*Intervention:* diet or physical activity related school policies either alone or as part of intervention programmes	*Intervention:* policy components which are insufficiently described to enable replication.
*Outcome:* body mass index (using valid reference curves to define overweight and obesity), body mass index z-score or standard deviation score, percentage of body fat, waist circumference, waist-to-hip ratio, waist-to-height ratio, skin pinch/skin fold thickness	*Outcome:* change in diet, physical activity or knowledge
*Context:* primary school or equivalent	*Context:* clinical settings
*Study design:* any experimental or observational study design (randomised controlled trial, controlled before and after study, interrupted time series, cohort study or cross-sectional study)	*Study design:* narrative reviews, editorials, opinions and letters, reports published as meeting abstracts only (where insufficient methodological details are reported to allow critical appraisal of study quality)
*Follow-up:* ≥6 months [21]	*Follow-up:* <6 months

Studies which evaluated national, regional and school specific policies related to diet or physical activity during primary education, including multifaceted interventions which included a policy component using an anthropometric outcome were considered eligible. Given that policies are unlikely to be introduced experimentally with controls, controlled before and after studies and interrupted time series, cohort and cross-sectional studies were considered eligible as well as randomised controlled trials. A minimum follow-up or exposure to the policy was set at six months in line with the National Institute for Health and Clinical Excellence Obesity guidance [21].

#### Study identification

Having removed any duplicates using reference management software all article titles were screened by AJW. The resulting titles and abstracts were independently assessed for eligibility by AJW and AJH and the full texts of all potentially eligible articles were retrieved for independent review. Any disagreements were resolved by discussion. Articles deemed eligible went on to data extraction and quality assessment.

#### Data extraction and quality assessment

The following data were extracted from each eligible article: study design; geographic location of study (country); source of funding; ethics approval; recruitment; summary characteristics of the study population; details of the intervention (policy name, target and any assessment of uptake); treatment of any control group; definition of obesity; duration of follow-up/exposure; and results. Standard tools were used to assess the quality of the studies [22-24]. The data extraction and quality assessment tool was piloted for suitability on four papers by AJW and AJH. Data extraction and quality assessment were undertaken by AJW and checked by AJH or KMW, any disagreement was resolved through discussion. Information was also extracted on whether and which stakeholders were involved in the development and implementation and whether the policy engaged families. Further details on three of the policies was sought (e.g. manuals, policy criteria) to identify the policy components [25-27].

#### Data analysis

As diet and physical activity are distinct concepts, an overall meta-analysis was not considered to be appropriate, instead policies which sought to affect similar behaviours, such as nutrition guidelines, were clustered and analysed. Standardised mean difference (Cohen’s *d*) in body mass index standard deviation score (BMI-SDS), were calculated for each study using standard calculations and the R package MAd [28-30]. As there is a positive bias in Cohen’s *d*-values calculated from studies with small sample sizes, effect sizes were adjusted into Hedges’ *g*-values [28-30].

Where studies reported multiple comparisons within each cluster (i.e. for girls and boys or for multiple time-points), we first calculated Hedges’ *g* for each comparison separately. Where a study did not report the combined effect and variance, we calculated the weighted mean of the multiple effects [28]. Where necessary the covariate outcome correlation or multiple correlation among studies using independent samples was assumed to be 0.3 (r = 0.3), whereas the correlation between pre- and post-scores was assumed to be 0.6 (r = 0.6). These assumptions were tested with sensitivity analysis reported in Additional file 2.

For studies that did not account for the potential clustering within schools, prior to effect size calculation, we divided the reported sample size by a ‘design factor’ (1 + [(m-1) × ICC]), where ‘m’ is the average number of participants in each school and ‘ICC’ is the intra-cluster correlation [17]. An ICC of 0.01 was chosen based on the findings of Johnson, *et al.*[17,31]. Further details on the calculation of Cohen’s *d*-values prior to adjustment into Hedges’ *g* and the combination of effect sizes can be found in Additional file 3.

Random effects meta-analysis of each cluster was undertaken in Stata [32] to obtain pooled estimates of the effect of each policy cluster. We quantified the extent to which the between-study variability observed was due to true between-study differences (rather than to chance) using the I^2^ statistic [28].

## Results

### Identified studies

**Figure 1:**
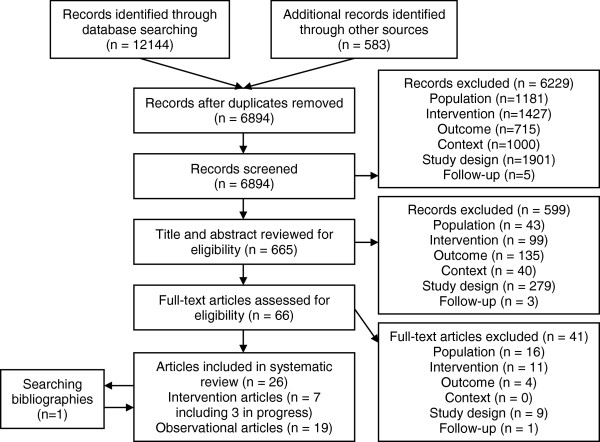
**PRISMA Flow diagram **[32]** of the identification of literature for inclusion in this systematic review.**

The study identification process and reasons for exclusion are illustrated in Figure 1[33]. A total of 6894 unique records were retrieved from the database and grey literature searches. Through the process of screening, title and abstract review and full text assessment, 25 articles were identified as potentially eligible for inclusion. Examining the bibliographies of these articles identified one additional paper [34]. These articles reported on 24 studies, three of which had yet to publish any results and, consequently, could not be included in the analysis [35-37]. The remaining 21 eligible studies are summarised in Table 2.

**Table 2:** Summary study characteristics

**Study/ Location**	**Study design/ Sample size**	**Policy**	**Gender**	**Age/ Follow-up or exposure duration**	**Ethnicity**	**Socioeconomic status**	**Baseline weight status**	**Outcome measure(s)/ Growth reference**
**Diet policies**								
Foster, *et al.* 2008 [55]/ USA	Randomised controlled trial/ n = 844, I:n = 479, C: n = 365	School nutrition policy initiative	I: 45.0% males, 55.0% females, C: 47.8% males, 52.2% females	I: mean ± SD 11.1 ± 1.0 years, C: mean ± SD 11.2 ± 1.0 years/ 2 years	I: 44.3% black, 22.4% Hispanic, 17.1% Asian, 10.7% white, 5.5% other, C: 46.8% black, 27.7% Asian, 14.2% white, 5.8% Hispanic, 5.5% other	Not described	I: 17.2% overweight, 25.34% obese, C: 16.5% overweight, 21.8% obese	BMI-SDS, overweight, obese/ CDC 2000
Baxter, *et al.* 2009 [53]/ USA	Cohort study/ n = 1,557	Location of School Breakfast Program consumption	Males and females	9–10 year olds/ 4 years	90% black	Not described	Not provided	BMI%
Henry, 2006 [50]/ USA	Cohort study/ n = 7,446	National School Lunch Program	Males and females	4–10 year olds/ 3 years	66% white, 26% African American, 5% Hispanic, 4% American Indian, 3% Asian	26% eligible for FSM	Kindergarten: 4% overweight, 4% obese, 3^rd^ grade: 4% overweight, 6% obese	Overweight from BMI/ CDC2000
Hernandez, Francis and Doyle, 2003 [41]/ USA	Cohort study/ n = 1,140	National School Lunch Program	50% males, 50% females	Mean ± SD 6.2 ± 0.4 years/ 9 years	54% white, 24% Hispanic, 12% black, 10% other	37% household income < $20,000	Mean BMI% ± SD, Kindergarten: 63.3 ± 28.0, 1^st^ grade: 62.1 ± 29.8, 3^rd^ grade: 66.6 ± 28.8, 5^th^ grade: 69.4 ± 28.7	BMI/ CDC 2000
Hinrichs, 2010 [47]/ USA	Cohort study/ n = 130,353	National School Lunch Program	47.4% males, 52.6% females	Not provided (studied adults who had participated in policy during childhood)	88.0% white, 10.3% black, 1.6% other	Not described	Males: 42.5% overweight, 8.0% obese, Females: 22.4% overweight, 7.4% obese	BMI, overweight, obese
Millimet, Tchernis and Husain, 2008 [43] and 2010 [44]/ USA	Cohort study/ n = 13.531	National School Lunch Program and School Breakfast Program	50.7% males, 49.3% females	Mean ± SD 9.2 ± 0.4 years/ 3 years	57.9% white, 17.4% Hispanic, 13.8% black, 4.5% Asian	Mothers education: 19.8% high school, 28.1% some college, 14.4% bachelor’s degree, 8.4% advanced college degree	Kindergarten: 25.8% overweight, 11.4% obese, 3^rd^ grade: 32.5% overweight, 17.1% obese	BMI%, BMI growth rate/ CDC 2000
Millimet and Tchernis, 2009 [42]/ USA	Cohort study/ n = 7,824	School Breakfast Program	51.3% males, 48.7% females	Mean ± SD 9.1 ± 0.3 years/ 5 years	55.4% white, 19.1% Hispanic, 13.7% black	Mean socioeconomic status index 0.06 ± 0.77	3^rd^ grade: 36.5% overweight or obese, 5^th^ grade: 41.4% overweight or obese	BMI growth rate/ CDC 2000
Ramirez-Lopez, *et al.* 2005 [54]/ Mexico	Cohort study/ n = 360, I: n = 254, C: n = 106	School Breakfast Program	Males and females	I: mean ± SD 8.6 ± 1.3 years, C: mean ± SD 8.4 ± 1.3 years/ 9 months	Not described	Not described	I: 10.6% overweight, 10.6% obese, C: 8.5% overweight, 11.3% obese	BMI, body fat%, overweight, obese/ CDC 2000
Fox, *et al.* 2009 [56]/ USA	Cross-sectional study/ n = 706	Nutrition guidelines	51% males, 49% females	Mean 8.8 years/ > 1 year	52% white, 24% Hispanic, 17% black, 7% other	48.49% eligible for FSM	Not described	BMI-SDS, obese/ CDC 2000
Jones, *et al.* 2003 [34]/ USA	Cross-sectional study/ n = 772	National School Lunch Program and School Breakfast Program	50% males, 50% females	50% aged 5–8 years, 50% aged 9–12 years/ up to 7 years	58.2% Black, 25,8% white, 10.4% Hispanic, 0.1% other	Head of household has <12 years education 33.9%, household food insecure 24.0%	34.2% overweight or obese	Overweight and obese from BMI%/ CDC 2000
**Physical activity policies**								
Donnelly, *et al.*2009 [62]/ USA	Randomised controlled trial/ n = 1,527, I: n = 814, C: n = 713	Physical activity across the curriculum	48.8% males, 51.2% females	7–9 year olds/ 3 years	77.4% Caucasian, 10.1% Hispanic, 6.2% African American, 3.6% multi-ethnic, 1.6% Native American, 1.2% Asian	43% eligible for FSM	Mean BMI ± SD I: 17.9 ± 3.1, C:18.0 ± 3.7	BMI/ CDC 2000
Heelan, *et al.* 2009 [61]/ USA	Controlled before and after study/ n = 324, I: n = 201, C: n = 123	Walking school bus scheme	44.8% males, 55.2% females	Mean ± SD I: 8.1 ± 1.7 years, C: 8.4 ± 1.6 years/ 2 years	90% white, 7% Hispanic, 3% other	~30% eligible for FSM	Mean BMI% ± SD I:67.6 ± 22.3, C:61.6 ± 29.1	BMI-SDS,% body fat/ CDC 2000
Chiodera, *et al.*2008 [60]/ Italy	Cohort study/ n = 4,500	Professionally led PE	51.1% males, 48.9% females	6–10 year olds/ 8 months	Not described	Not described	Mean BMI ± SD: grade 1 16.3 ± 2.3, grade 2 16.9 ± 2.5, grade 3 17.2 ± 2.6, grade 4 17.9 ± 3.1, grade 5 18.6 ± 3.1	BMI
Datar and Sturm, 2004 [38]/ USA	Cohort study/ n = 9,751, I: n = 8,917, C: n = 834	Increased PE duration of 1 hour per week	50% males, 50% females	4–6 year olds/ 1 year	I: 61% white, 16% Hispanic, 12% black, 11% other, C: 58% white, 20% black, 15% Hispanic, 8% other	I: 13% family income < $15,000, C: 16% family income < $15,000	I: 15% overweight, 11% obese, C: 15% overweight, 12% obese	BMI/ CDC 2000
Fernandes, 2010 [39] and Fernandes and Sturm, 2011 [40]/ USA	Cohort study/ n = 8,246	Meeting the National Association for Sport and Physical Education (NASPE) guidelines	50.4% males, 49.6% females	6–11 year olds/ 5 years	61.2% white, 18.7% Hispanic, 13.1% black, 7.0% other	11.3% below the poverty threshold	Mean BMI% ± CD 60.8 ± 28.3, 13.3% obese	BMI%/ CDC 2000
**Combined policies**								
Johnson, *et al.* 2012 [31]/ Australia	Controlled before and after study/ n = 1318	Be Active Eat Well	I: 46.3% males, 53.7% females, C: 50.8% males, 49.2% females	Baseline mean ± SD – I: 8.16 ± 2.25, C: 8.19 ± 2.15. Follow-up mean ± SD – I: 11.1 ± 2.26 C:10.3 ± 2.14	Parents born overseas [27]* I: 6% C: 12%	Mothers didn’t complete high school education I: 47.1% C: 40.6%	Baseline mean BMI-SDS ± SD – I: 0.59 ± 0.92, C: 0.60 ± 0.87. Follow-up mean BMI-SDS ± SD – I: 0.54 ± 0.94 C:0.59 ± 0.88	BMI-SDS/ CDC 2000
Jordan, *et al.* 2008 [64]/ USA	Controlled before and after study/ n = 577	Utah’s Gold Medal Schools	I: 51% males, 49% females, C:52% males, 48% females	Mean ± SD I: 9.0 ± 1.6 years, C: 9.0 ± 1.6 years/ 1 year	I: 85.8% white, 7.6% Hispanic, 2.8% Hawaiian, 0.7% Asian, 0.4% American Indian, 0.0% African American, 2.8% other C: 86.7% white, 7.0% Hispanic, 2.1% African American, 0.7% American Indian, 0.7% Asian, 0.4% Hawaiian, 2.5% other	Maternal education: <high school I: 1.7%, C: 4.9%, high school graduate I: 19.9%, C: 25.9%, some college I: 41.9%, C: 41.6%, college graduate I: 32.0%, C: 25.5%, graduate degree I: 4.5%, C: 2.1%	Not described	BMI-SDS/ CDC 2000
Chomitz, *et al.* 2010 [63]/ USA	Cohort study/ n = 1,858	Healthy living Cambridge kids	51.8% males, 48.2% females	Mean ± SD 7.7 ± 1.8 years/ 3 years	37.3% white, 36.9% black, 14.0% Hispanic, 10.2% Asian, 1.7% other	43.3% from low income families	Mean BMI-SDS ± SD 0.7 ± 1.1. 16.8% overweight, 20.2% obese	BMI-SDS, overweight, obese/ CDC 2000
Harrison, *et al.* 2011 [57]/ UK	Cross-sectional study/ n = 1,724	Variety of diet and physical activity related policies	44.4% males, 55.6% females	Mean ± SD 10.3 ± 3.1 years/ 5 years	Not described	Age parent left full time education: <16 years 46.5%, 16–18 years 33.4%, >18 years 20.1%	16.8% overweight, 5.2% obese	Fat mass index (FMI)/ IOTF
Veugelers and Fitzgerald, 2005 [58]/ Canada	Cross-sectional study/ 279 schools	Nutrition policy and Annapolis valley health promoting schools project	Males and females	10–11 year olds/ 5 years	Not described	Not described	32.8% overweight, 9.9% obese	Overweight, obese from BMI-SDS/ IOTF
Zhu, *et al.* 2010 [59]/ USA	Cross-sectional study/ 738 schools	Variety of diet and physical activity related policies	Males and females	Not described/ up to 6 years	Not described	53% eligible for FSM	Mean ± SD 71.7% ± 12.6 within BMIHFZ	BMIHFZ

### Study characteristics and quality

Ten studies examined diet related policies, five physical activity related policies, and six examined policies with both diet and physical activity related components (henceforth known as combined policies) (Table 2). Despite the lack of time restrictions on the searches all the included studies had been published since 2003. Sixteen of the studies took place in the USA with the remaining five taking place in: Australia, Canada, Italy, Mexico, and the UK. The 21 studies employed the following study designs: randomised controlled trial (2 studies), controlled before and after study (3 studies), cohort study (11 studies) and cross-sectional study (5 studies). Five of the cohort studies analysed data from the Early Childhood Longitudinal Survey – Kindergarten (ECLS-K), these studies are easily identifiable in the forest plots, and no more than two studies using this cohort are ever combined [38-44].

All the included studies examined BMI as an outcome categorised as overweight or obese, or adjusted to standard deviation scores (BMI-SDS), percentiles (BMI%), growth rates or Healthy Fitness Zone (BMIHFZ) [45]. The Healthy Fitness Zone is another categorisation of BMI-SDS like overweight and obesity associated with body fat and therefore could be analysed like overweight and obesity [45]. Additional outcome measures included: fat mass index (FMI), body fat percentage, waist-hip ratio and waist-height ratio, however, these outcomes were only reported by a small number of studies and therefore were not meta-analysed. As overweight and obesity are cut-points along the scale of BMI-SDS, odds ratios were converted to effect sizes following the method detailed by Chinn [46]. When studies reported BMI both continuously and categorically, these results were combined using the methods outlined by Borenstein, *et al.*[28]. One study reported unadjusted BMI as an outcome as the subjects were adults who had been exposed to the USA National School Lunch Program (NSLP) as children, this study was excluded from the meta-analysis [47].

Study quality is summarised in Table 3. Of the observational studies, the majority utilised a sample which was representative or somewhat representative of the population. Twelve of the studies adjusted the results for socioeconomic status, ethnicity or additional factors. All except one study assessed the outcome independently from the assessment of exposure, and all studies had sufficient follow-up duration. Seven observational studies lost less than 20% of the sample during the study, however, five studies experienced loss to follow-up at a level which may have introduced bias.

**Table 3:** Summary of study quality

**Randomised controlled trials**	**Random allocation**	**Baseline measurement**	**Reliability of outcome measure**	**Blinding**	**Adequacy of follow-up**	**Protection against contamination**
Donnelly, *et al.* 2009 [62]	Completed	Completed	Not clear	Completed	Adequate	Completed
Foster, *et al.* 2008 [55]	Completed	Completed	Sufficient	Not blinded	Significant loss to follow-up	Completed
**Controlled before-after studies**	**Second site control**	**Baseline measurement**	**Reliability of outcome measure**	**Blinding**	**Adequacy of follow-up**	**Protection against contamination**
Heelan, *et al.* 2009 [61]	Sufficient	Completed	Sufficient	Not clear	Significant loss to follow-up	Completed
Johnson, *et al.* 2012 [31]	Sufficient	Completed	Sufficient	Not done	Adequate	Completed
Jordan, *et al.* 2008 [64]	Sufficient	Not clear	Not clear	Not clear	Significant loss to follow-up	Completed
**Cohort Studies**	**Representativeness of the cohort/sample**	**Comparability of cohorts**	**Ascertainment of exposure**	**Assessment of outcome**	**Duration of exposure**	**Adequacy of exposure**
Baxter, *et al.* 2009 [53]	Not described	Not indicated	Measured as part of the study	Independent of exposure	Sufficient	No statement
Chiodera, *et al.* 2008 [60]	Representative	Did not control for socioeconomic status or ethnicity	Measured as part of the study	Independent of exposure	Sufficient	Sufficient
Chomitz, *et al.* 2010 [63]	Somewhat representative	Controlled for ethnicity, socioeconomic status and additional factors	Measured as part of the study	Independent of exposure	Sufficient	Subjects lost to follow-up unlikely to introduce bias
Datar and Sturm, 2004 [38]	Representative	Controlled for ethnicity, socioeconomic status and additional factors	Measured as part of the study	Independent of exposure	Sufficient	Subjects lost of follow-up may have introduced bias
Fernandes, 2010 [39] and Fernandes and Sturm, 2011 [40]	Representative	Controlled for ethnicity, socioeconomic status and additional factors	Structured interview	Independent of exposure	Sufficient	Subjects lost to follow-up may have introduced bias
Henry, 2006 [50]	Somewhat representative	Did not control for socioeconomic status or ethnicity	Measured as part of the study	Independent of exposure	Sufficient	Sufficient
Hernandez. Francis and Doyle, 2011 [41]	Representative	Controlled for ethnicity, socioeconomic status and additional factors	Written self report	Independent of exposure	Sufficient	Sufficient
Hinrichs, 2006 [47]	Representative	Controlled for ethnicity and, socioeconomic status	Measured as part of the study	Independent of exposure	Sufficient	No statement
Millimet and Tchernis, 2009 [42]	Somewhat representative	Controlled for ethnicity, socioeconomic status and additional factors	Measured as part of the study	Independent of exposure	Sufficient	Subjects lost to follow-up may have introduced bias
Millimet, Tchernis and Husain, 2008 [43] and 2010 [44]	Representative	Controlled for ethnicity, socioeconomic status and additional factors	Measured as part of the study	Independent of exposure	Sufficient	Sufficient
Ramirez-Lopez, *et al.* 2005 [54]	Somewhat representative	Controlled for some factors but not socioeconomic status or ethnicity	Measured as part of the study	No description	Sufficient	Subjects lost to follow-up may have introduced bias
**Cross-sectional Studies**	**Representativeness of the cohort/sample**	**Comparability of cohorts**	**Ascertainment of exposure**	**Assessment of outcome**	**Duration of exposure**	**Adequacy of exposure**
Fox, *et al.* 2009 [56]	Somewhat representative	Controlled for ethnicity, socioeconomic status and additional factors	Measured as part of the study and structured interviews	Independent of exposure	Sufficient	Sufficient
Jones, et al. 2003 [34]	Somewhat representative	Controlled for ethnicity and, socioeconomic status	Measured as part of the study	Independent of exposure	Sufficient	Sufficient
Harrison, et al. 2011 [57]	Somewhat representative	Controlled for socioeconomic status	Measured as part of the study	Independent of exposure	Sufficient	Subjects lost to follow-up may have introduced bias
Veugelers and Fitzgerald, 2005 [58]	Somewhat representative	Controlled for socioeconomic status and additional factors	Measured as part of the study	Independent of exposure	Sufficient	Sufficient
Zhu, et al. 2010 [59]	At risk group	Did not control for socioeconomic status or ethnicity	Measured as part of the study	Independent of exposure	Sufficient	Subjects lost of follow-up unlikely to introduce bias

Due to the nature of policy interventions, the randomised controlled trials and controlled before and after studies could not meet some of the quality criteria generally applied to these study designs. Blinding of the outcome assessment may not always have been possible and, for some studies, loss to follow up was greater than 20%. However, each study employed a valid design, in terms of the use of second sites as controls, random allocation and protection from contamination.

### Participant characteristics

The demographics and baseline weight status of the participants of each study are listed in Table 2. All the studies assessed both males and females and in those studies that reported gender distribution there was approximately equal numbers of each sex. Thirteen of the studies examined children across the age span of primary education, while four studies examined children towards the end of primary education, one study examined children in the middle of primary education and three studied those beginning primary education. Five of the studies did not report ethnicity data. Of the sixteen studies which did, eleven had a sample which was majority white, two studies had a majority black, two studies without a majority ethnic group had black as the largest minority and one study only reported that the majority of participants' parents were natives. Fifteen of the studies reported the socioeconomic status of the participants using a variety of measures which are reported in Table 2.

Fifteen studies utilised the Center for Disease Control and Prevention (CDC) 2000 BMI reference categories and an additional paper appears to have used this categorisation but did not report it [48]. Two studies utilised the International Obesity Task Force (IOTF) BMI reference categories [49]. Of the three remaining studies one categorised BMI according to the Healthy Fitness Zone [45], one studied adults who had been exposed to the NSLP as children and the remaining paper did not report which reference was utilised. The majority of studies reported that at baseline between 20% and 40% of the sample was overweight or obese, however, Henry [50] reported a prevalence of overweight and obesity below 10%. Three studies did not report the baseline weight characteristics of the participants.

### Study results

Key results from each study are presented in Table 4. Alongside the quantitative results, the results have also been depicted symbolically to aid understanding in a similar way to that described by McCartney, *et al.*[51] and Thomson [52]. Table 4 contains columns for each of the outcomes assessed and within each column is a symbol. If the symbol is a dash (−) that outcome was not assessed by the study, otherwise the direction of the arrow indicates the direction of the association (↑; positive, ↓; negative, ↕; mixed, ↔; no effect), black arrows indicate significant (p < 0.05) results, while grey arrows are non-significant.

**Table 4:** Policy summaries and results

**Study**	**Involvement**		**Policy components**	**Impact†**					**Results**	**Sig**
	Stakeholder*	**Family**		**BMI-SDS**	**Ov**	**Ob**	**BF**	**BMIHFZ**	**Statistic (95% confidence interval) unless otherwise stated**	
**Diet policies**										
Foster, *et al.* 2008 [55]	✓^a,c,e,f,h^	✓	School nutrition policy initiative				**-**	**-**	Adjusted change in BMI-SDS -0.01 (-0.08,0.06)	
									Adjust odds ratio overweight 0.65 (0.54,0.79)	Sig
									Adjusted odds ratio obesity 1.09 (0.85,1.40)	
Baxter, *et al.* 2009 [53]			Location of School Breakfast Program consumption		**-**	**-**	**-**	**-**	Δ mean BMI% breakfast in classroom compared to the cafeteria 2.64 (p=0.06)	
Henry, 2006 [50]			National School Lunch Program	**-**		**-**	**-**	**-**	Hedges’ *g* overweight 1.39 (0.55,2.24)	Sig
Hernandez, Francis and Doyle, 2003 [41]			National School Lunch Program		**-**	**-**	**-**	**-**	Adjusted change in BMI Kindergarten: 0.12 (-0.33,0.57)	
									Adjusted change in BMI 1^st^ grade: 0.20 (-0.29,0.69)	
									Adjusted change in BMI 3^rd^ grade: 0.36 (-0.25,0.97)	
									Adjusted change in BMI 5^th^ grade: 0.52 (-0.24,1.28)	
Hinrichs, 2010 [47]			National School Lunch Program				**-**	**-**	Adjusted change in BMI ♂ -0.02 (-0.06,0.02), ♀ -0.02 (-0.07,0.03)	
									Change in prevalence of overweight ♂ <-0.01 (-0.01,<0.01), ♀ <-0.01 (-0.01, <0.01)	
									Change in prevalence of obesity ♂ <-0.01 (<-0.01, <0.01), ♀ <-0.01 (<-0.01, <0.01)	
Millimet, Tchernis and Husain, 2008 [43] and 2010 [44]									Bivariate Probit results assuming ρ=0.1	
			National School Lunch Program,	**-**			**-**	**-**	Change in probability of being overweight 0.13 (0.07, 0.20)	
									Change in probability of being obese 0.13 (0.05, 0.20)	
			School Breakfast Program	**-**			**-**	**-**	Change in probability of being overweight -0.07 (-0.14, <-0.01)	
									Change in probability of being obese -0.05 (-0.13, 0.03)	
Millimet and Tchernis, 2009 [42]									Bias corrected minimum bias estimator assuming θ=0.25	
			School Breakfast Program				**-**	**-**	Change in BMI growth rate 3^rd^ grade: -0.03 (-0.06, <-0.01)	
									Change in probability of overweight 3^rd^ grade: -0.21 (-0.33, -0.03)	
									Change in probability of obesity 3^rd^ grade: -0.17 (-0.26, -0.01)	
									Change in BMI growth rate 5^th^ grade: -0.04 (-0.08, 0.01)	
									Change in probability of overweight 5^th^ grade: -0.28 (-0.40, -0.09)	
									Change in probability of obesity 5^th^ grade: -0.12 (-0.28, -0.04)	
Ramirez-Lopez, *et al.* 2005 [54]			School Breakfast Program					**-**	Change in BMI Intervention: 0.1, Control: -0.1	
									Change in BF% Intervention: -0.2, Control: -0.5	
									Change in prevalence of overweight or obesity Intervention: 1, Control: -1	
									Change prevalence of obesity Intervention: 1, Control:-3	
Fox, *et al.* 2009 [56]			À la carte LNED food not available		**-**		**-**	**-**	Adjusted change in BMI-SDS -0.15 (-0.37,0.07)	
									Adjusted odds ratio obesity 1.09 (0.57,2.08)	
			Milk not available for school lunch		**-**		**-**	**-**	Adjusted change in BMI-SDS -0.13 (-0.33,0.07)	
									Adjusted odds ratio obesity 1.17 (0.75,1.82)	
			Fresh fruit/ raw vegetables available		**-**		**-**	**-**	Adjusted change in BMI-SDS 0.19 (0.01,0.37)	
									Adjusted odds ratio obesity 1.13 (0.73,1.75)	
			Fried potato products not available		**-**		**-**	**-**	Adjusted change in BMI-SDS 0.20 (<0.01,0.40)	
									Adjusted odds ratio obesity 2.70 (1.58,4.62)	Sig
			Desserts offered ≤once a week		**-**		**-**	**-**	Adjusted change in BMI-SDS 0.08 (-0.08,0.24)	
									Adjusted odds ratio obesity 1.78 (1.13,2.80)	Sig
Jones, *et al.* 2003 [34]									Adjusted odds ratio overweight and obesity:	
			National School Lunch Program	**-**			**-**	**-**	Food secure ♂1.06 (0.53,2.08), ♀0.49 (0.22,1.10),	
									Food insecure ♂0.62 (0.25,1.54), ♀0.29 (0.11,0.80)	Sig♀
			School Breakfast and National School Lunch Programs	**-**			**-**	**-**	Food secure ♂1.33 (0.81,2.18), ♀0.66 (0.35,1.26)	
									Food insecure ♂0.85 (0.42,1.74), ♀0.42 (0.19,0.96)	Sig♀
**Physical activity policies**										
Donnelly, *et al.*2009 [62]	✓^h^		Physical Activity Across the Curriculum				**-**	**-**	BMI Hedges’ *g* 0.01 (-0.09,0.11)	
Heelan, *et al.* 2009 [61]			Walking school bus scheme		**-**	**-**		**-**	Intervention vs. Control BMI-SDS Hedges’ *g*: -0.21 (-0.58,0.15)	
									Frequent v. passive BMI-SDS Hedges’ *g*: -0.49 (-0.94,-0.03)	Sig
									Infrequent v. passive BMI-SDS Hedges’ *g*: -0.17 (-0.61,0.28)	
									Intervention vs. Control BF% Cohen’s d: -0.25 (-0.61,0.11)	
									Frequent v. passive BF% Cohen’s d: -0.59 (-1.05,-0.13)	Sig
									Infrequent v. passive BF% Cohen’s d: -0.28 (-0.72,0.17)	
Chiodera, *et al.*2008 [60]			Professionally led PE		**-**	**-**	**-**	**-**	Change in BMI grade 1: -0.21	Sig
									Change in BMI grade 2: -0.05	
									Change in BMI grade 3: -0.06	
									Change in BMI grade 4: 0.04	
									Change in BMI grade 5: 0.02	
Datar and Sturm, 2004 [38]			Increased PE duration of 1 hour per week		**-**	**-**	**-**	**-**	Adjusted change in BMI, normal weight ♂ 0.04 (-0.04,0.12)	
									Adjusted change in BMI, normal weight ♀ 0.01 (-0.07,0.10)	
									Adjusted change in BMI, overweight or obese ♂ -0.07 (-0.19,0.05)	
									Adjusted change in BMI, overweight or obese ♀ -0.32 (-0.46,-0.17)	Sig
Fernandes, 2010 [39] and Fernandes and Sturm, 2011 [40]			Meeting the National Association for Sport and Physical Education (NASPE) guidelines		**-**	**-**	**-**	**-**	PE duration Adjusted change in BMI% -0.74 (-1.78,0.30), ♂ -1.56 (-3.03,-0.09), ♀ 0.05 (-1.40,1.50)	Sig♂
									Break period duration: adjusted change in BMI% -0.74 (-1.33,-0.15), ♂ -0.81 (-1.67,0.05), ♀ -0.69 (-1.49,0.11)	Sig
**Combined policies**										
Johnson, *et al.* 2012 [31]	✓^e,f,g,h^	✓	Be Active Eat Well		**-**	**-**	**-**	**-**	Adjusted change in BMI-SDS -0.085 (-0.18,0.01)	
			HE policy		**-**	**-**	**-**	**-**	Adjusted change in BMI-SDS -0.008 (-0.06,0.04)	
			PA policy		**-**	**-**	**-**	**-**	Adjusted change in BMI-SDS -0.006 (-0.06,0.05)	
Jordan, *et al.* 2008 [25,64]	✓^b,c,d,e,f,h^	✓	Utah’s Gold Medal Schools		**-**	**-**	**-**	**-**	Change in BMI-SDS Intervention: 0.21 (-0.71,1.13), Control: 0.53 (-0.21,1.27)	
Chomitz, *et al.* 2010 [63]	✓^c,e,f,h^	✓	Healthy Living Cambridge Kids				**-**	**-**	Change in BMI-SDS -0.04	Sig
									Change in prevalence of overweight 0.6% points	
									Change in prevalence of obesity -2.2% points	Sig
Harrison, *et al.* 2011 [57]			Cookery lessons	**-**	**-**	**-**		**-**	None of the policies were significantly associated with FMI in females, while only being able to eat any food at break times and being able to play 3-4 games during break times where association with higher FMI in males.	
			Foods permitted during break periods	**-**	**-**	**-**		**-**		
			HE policy	**-**	**-**	**-**		**-**		
			Sports allowed during break periods	**-**	**-**	**-**		**-**		
			‘Park and stride’ scheme	**-**	**-**	**-**		**-**		
			PA policy	**-**	**-**	**-**		**-**		
			PA and HE policy	**-**	**-**	**-**		**-**		
Veugelers and Fitzgerald, 2005 [26,58]			Nutrition policy	**-**			**-**	**-**	Adjusted odds ratio overweight: 0.91 (0.77,1.09)	
									Adjusted odds ratio obesity: 0.85 (0.63,1.55)	
	✓^a,c,d,e,f,g,h^	✓	Annapolis Valley Health Promoting Schools Project	**-**			**-**	**-**	Adjusted odds ratio overweight: 0.41 (0.32,0.53)	Sig
									Adjusted odds ratio obesity: 0.28 (0.14,0.57)	Sig
Zhu, *et al.* 2010 [59]	✓^a,b,d,f,g,h^		Professionally led PE	**-**	**-**	**-**	**-**		Adjusted change in BMIHFZ achievement rate 0.62 (0.01,1.23)	Sig
			Duration of PE periods	**-**	**-**	**-**	**-**		Adjusted change in BMIHFZ achievement rate 0.05 (-0.03,0.13)	
			Number of PE periods	**-**	**-**	**-**	**-**		Adjusted change in BMIHFZ achievement rate 1.06 (0.47,1.65)	Sig
			Duration of Break periods	**-**	**-**	**-**	**-**		Adjusted change in BMIHFZ achievement rate 2.71 (1.75,3.67)	Sig
			Number of break periods	**-**	**-**	**-**	**-**		Adjusted change in BMIHFZ achievement rate -2.25 (-3.86,-0.64)	Sig
			Cancel due to weather	**-**	**-**	**-**	**-**		Adjusted change in BMIHFZ achievement rate -1.26 (-3.73,1.21)	
			PE exemptions	**-**	**-**	**-**	**-**		Adjusted change in BMIHFZ achievement rate -0.34 (-0.65,-0.03)	Sig
			USDA	**-**	**-**	**-**	**-**		Adjusted change in BMIHFZ achievement rate 0.02 (-1.49,1.53)	
			Wellness council	**-**	**-**	**-**	**-**		Adjusted change in BMIHFZ achievement rate 0.41 (-0.04,0.86)	

*Abbreviations*: *♂* male, *♀* female, *BF%* body fat percentage, *BMI* body mass index, *BMI%* BMI percentile, *BMIHFZ* BMI Healthy Fitness Zone [45], *BMI-SDS* BMI standard deviation score, *FSM* free or reduced school meals, *HE* healthy eating, *LNED* low-nutrient, energy-dense, *PA* physical activity, *PE* physical education, *SD* standard deviation, *SE* standard error, *Sig* p < 0.05, *TV* television, *USDA* United States Department of Agriculture wellness program.

*Stakeholders: school administrators^a^, school board^b^, sports coaches^c^, food services^d^, health services^e^, parents^f^, pupils^g^, teachers^h^.

†Impact: a symbolic representation of the statistical results. **↑**: positive association, **↓**: negative association, **↕**: mixed association, **↔**: no association. Black arrows indicate significance (p < 0.05), while grey indicates non-significance (p > 0.05) [51,52].

### Diet related policies

Thirteen studies evaluated diet related policies, including five evaluating the NSLP [34,41,43,44,47,50] and five School Breakfast Program (SBP) [34,42-44,53,54] (two studies evaluated both the NSLP and SBP [34,43,44]). The NSLP and SBP were developed and implemented to improve the nutritional state and health of undernourished children [41,47]. Subsequently, unlike the general diet related policies, the NSLP and SBP are targeted at specific pupils, their intention not being to benefit the entire population of children, just an at risk group. With improved nutrition it has become a concern that the NSLP and SBP could be contributing to unhealthy weight gain [41]. The remaining five studies evaluated policies related to the availability of foods within schools, one of which did not present quantitative results and therefore only four studies could be pooled [31,55-58]. Due to the underlying conceptual differences in the intention and population of the NSLP, SBP and other diet related policies, the studies were separated into three policy groups for meta-analysis.

**Figure 2:**
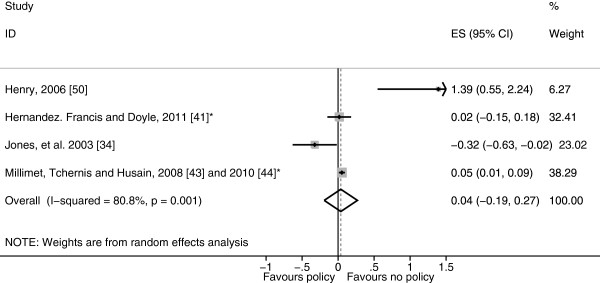
**Forest plot showing body mass index standard deviation score effect sizes (Hedges’ *g*) of studies evaluating participation in the National School Lunch Program.** *Study using the Early Childhood Longitudinal Study – Kindergarten (ECLS-K) cohort.

**Figure 3:**
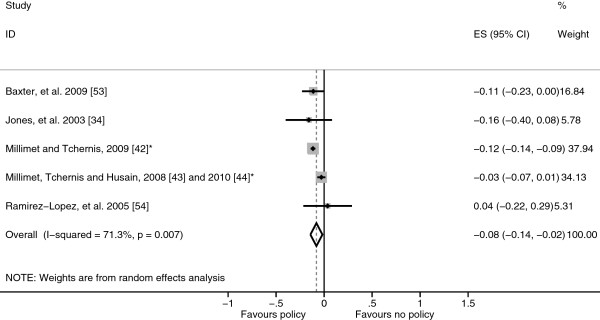
**Forest plot showing body mass index standard deviation score effect sizes (Hedges’ *g*) of studies evaluating participation in the School Breakfast Program.** *Study using the Early Childhood Longitudinal Study – Kindergarten (ECLS-K) cohort.

The pooled result of participation in the NSLP was a small non-significant rise in BMI-SDS (0.038 BMI-SDS, 95% confidence interval (95% CI) -0.193 to 0.269) (Figure 2). The study by Hinrichs [47] which could not be included in the meta-analysis resulted in a similar non-significant difference in BMI, overweight or obesity status between adults who had and hadn’t participated in the NSLP. The pooled result of the five studies that evaluated the SBP was a significantly lower BMI-SDS among those who participated in the SBP (−0.080 BMI-SDS, 95% CI −0.143 to −0.017) (Figure 3). However, it should be noted that there was a significant degree of heterogeneity in both of these clusters (Figures 2 and 3).

**Figure 4:**
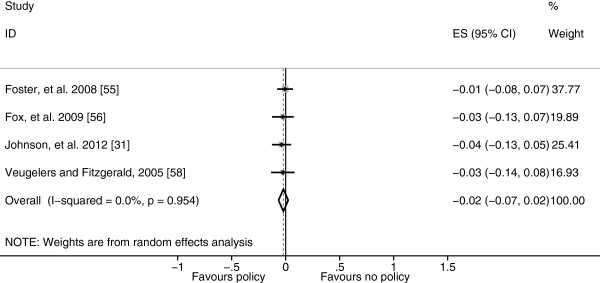
**Forest plot showing body mass index standard deviation score effect sizes (Hedges’ *g*) of studies evaluating other diet related policies.**

The other diet related policies evaluated included: removing low nutrient, energy-dense foods, fried potato products, desserts and whole or 2% milk from cafeterias, ensuring fruits and vegetables are available in the cafeteria, children being prevented from eating any food at break periods and attending a school with a nutrition policy which enabled children to choose healthier foods [31,56-58]. The pooled effect of these diet related policies was a small and non-significant reduction of −0.021 BMI-SDS (95% CI −0.066 to 0.023) (Figure 4).

### Physical activity related policies

**Figure 5:**
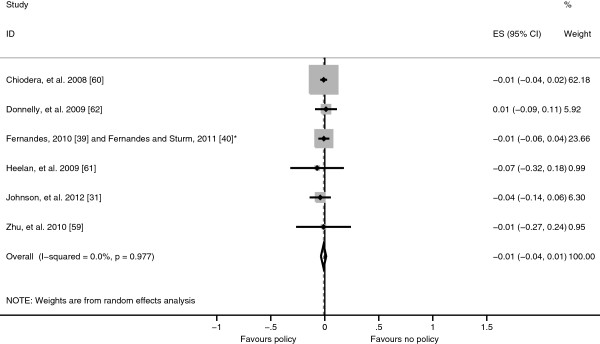
**Forest plot showing body mass index standard deviation score effect sizes (Hedges’ *g*) from studies evaluating physical activity related policies.** *Study using the Early Childhood Longitudinal Study – Kindergarten (ECLS-K) cohort.

Eight studies examined physical activity related policies, two of which could not be included in the meta-analysis as they did not provide sufficient information for the calculation of effect sizes [31,38-40,57,59-62]. The policies evaluated included: having a general physical activity policy, the use of qualified PE teachers, PE and break period duration and frequency, variety of activities permitted during break periods, the number of valid reasons for exemption from PE, cancelling PE due to the weather, physical activity incorporated into lessons and active commuting schemes [31,38-40,57,59-62]. The pooled effect of all policies related to physical activity was a small and non-significant reduction in BMI-SDS (−0.011, 95% CI −0.036 to 0.013) (Figure 5).

### Combined policies

**Figure 6:**
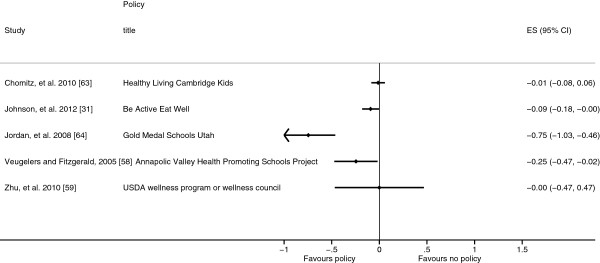
**Forest plot showing body mass index standard deviation score effect sizes (Hedges’ *g*) from studies evaluating the combined policies.**

Six studies evaluated policies with both diet and physical activity related components, one of which did not report the quantitative results just the direction and significance and therefore effect sizes could not be calculated [57-59,63,64]. Four of these studies also considered policy components which related to either diet or physical activity separately and these components have been included in the respective clusters discussed above [31,57-59]. Five of the policies were components of multifaceted intervention programmes, subsequently there was great variability in the nature of the combined policies leading to high heterogeneity (I^2^ = 85.1%) and the effects of these policies have not been combined. However, the individual results and effect sizes are detailed in Table 4 and Figure 6. Harrison, *et al.*[57] reported a non-significant association between having policies promoting both physical activity and healthy eating and FMI. Similarly, the USDA wellness program or wellness council was not significantly associated with an improvement in the proportion of children within the BMIHFZ in the study by Zhu, *et al.*[59]. Whereas, exposure to Healthy Living Cambridge Kids was significantly associated with lower BMI-SDS and prevalence of obesity, but not the prevalence of overweight which resulted in a non-significant combined effect size [63]. Chomitz, *et al.*[63] considered this result to be expected as obese children become overweight before reaching a healthy weight and therefore the prevalence of overweight might not change significantly. Participants in Gold Medal Schools programme in Utah, USA and Be Active Eat Well in Victoria, Australia gained less weight than control participants (Figure 6) [31,64]. The Annapolis Valley Health Promoting Schools Program (AVHPSP), evaluated by Veugelers and Fitzgerald [58], was significantly associated with reduced odds of both overweight and obesity. All four of these policies included significant stakeholder involvement within the development and implementation of the policy and engaged families.

## Discussion

The aim of this systematic review was to examine the effect of school diet and physical activity related policies upon anthropometric outcomes among children aged 4–11 years. Twenty-one studies were identified which examined a range of policies which were clustered as either diet related or physical activity related or both (combined policies) for analysis. Within the diet related policies cluster, eight studies evaluated the NSLP and SBP and as these policies target a subset of the population they were analysed separately from the other diet related policies. The NSLP was associated with a non-significant rise in BMI-SDS results, whereas the SBP was associated with a significant decrease in BMI-SDS and the other diet related policies were associated with a non-significant decrease in BMI-SDS, however, significant heterogeneity remained in the NSLP and SBP sub-clusters reducing the validity of these results (Figures 2, 3 and 4). Physical activity related policies were not associated with significant changes in BMI-SDS (Figure 5). Among the combined policies there was significant heterogeneity preventing meta-analysis, yet the combined policies demonstrated promising results in particular Gold Medal Schools, AVHPSP and Be Active Eat Well (Figure 6) [31,58,64]. These were multifaceted intervention programmes, which had wider health promotion aims, as well as improving diet and increasing physical activity. As well as utilising policy these programmes included stakeholder involvement and family engagement, methods recommended by Khambalia, *et al.*[16] as important components in school based obesity prevention interventions [25,26,31]. Gold Medal Schools also included health surveys and promotion among the school staff [25]. Five of the studies also evaluated the effect of the policy upon prevalence of underweight and none of the policies were found to have a negative impact, with some reporting a reduced prevalence of underweight among those exposed to the policy [34,42,47,55,63].

A strength of this review was the broad search strategy. School policy evaluation may be reported by a variety of disciplines and inside and outside of peer-reviewed journals and therefore through the variety of databases searched, the grey literature search and the inclusion of literature such as dissertations all the relevant studies were sought. Primarily, this demonstrated that there is a paucity of scientific evaluations of school policies as only 21 eligible studies were identified from the 6,894 retrieved, yet among the eligible studies where were a variety of designs, quality and policies which impinge upon the review. Among the 21 studies reviewed only five utilised experimental study designs which prevented the consideration of causal pathways in this review (Table 3). Loss to follow-up which may have led to bias was a significant concern for a number of the included studies as overweight or obese children may have been more likely to avoid follow-up (Table 3). As well as these differences in terms of quality and design, even when only the studies which evaluated similar policies were pooled for analysis there still remained significant heterogeneity. The length of follow-up/exposure within the included studies ranged from 8 months to more than 9 years (Table 2). The results of those studies with shorter follow-up/exposure duration may reflect the novelty of the policy or that insufficient time had passed for changes in body mass to be observed. The results from studies with longer follow-up/exposure reflect whether the policy prompted maintained behaviour change, or had only produced short lived changes in behaviour, which might also have contributed to the heterogeneity. Heterogeneity in the combined policies cluster was expected as there were differences in the policy each study evaluated, but the high heterogeneity in the NSLP and SBP clusters is unexpected and may be due to the differences in the sample characteristics or analytical methods (Table 2). Henry [50] reported a low baseline prevalence of overweight and obesity and produced an unusually large effect size, however, removing this result from the NSLP cluster only reduced the heterogeneity to I^2^ = 65.6%. Millimet and Tchernis [42] and Millimet, Tchernis and Husain [43] used complex analytical methods to account for non-random selection into the NSLP and SBP which may have produced greater differences between the studies. It is therefore more appropriate to understand the results of the meta-analyses presented as averages of the individual study effects rather than estimates of the common policy effect [65].

In order to calculate effect sizes, assumptions about the outcome correlations in studies using independent and non-independent samples were made; these assumptions were relaxed in a sensitivity analysis, reported in Additional file 2. However, there were no significant changes in the results. Combining continuous and categorical BMI-SDS outcomes also require some discussion. Foster, *et al.*[55] and Chomitz, *et al.*[63] both found the effect of the policy they evaluated to be inconsistent across weight categories which resulted in non-significant effect sizes. However, they found conflicting differences, Foster, *et al.*[55] found a significant effect in the overweight but not obese while Chomitz, *et al.*[63] found the opposite. Foster, *et al.*[55] argue that obesity is more intractable than overweight, supporting the need for early intervention to improve the weight status of overweight pupils before they become obese. While Chomitz, *et al.*[63] argue that the number of obese pupils becoming overweight may equal the number of overweight pupils obtaining a healthy weight resulting in no significant change in the prevalence of overweight. Rappaport, Daskalakis and Sendecki [66] recently re-evaluated the School Nutrition Policy Initiative evaluated by Foster, *et al.*[55] using routinely collected data and found the policy to no longer have an effect on either overweight or obesity. Repeating the meta-analysis replacing the results of Foster, *et al.*[55] with those of Rappaport, Daskalakis and Sendacki [66] did not significantly alter the results (Additional file 4). Ideally, policies would result in lowering the prevalence of both overweight and obesity which is likely to result in reduced mean BMI-SDS, suggesting that combining the results was appropriate [31,67].

This review evaluated the effect of school policies upon an objective measure of weight status (BMI-SDS) unlike previous reviews which have evaluated physical activity and diet outcomes, which may be more subjective [11-16]. Therefore, the positive effects of school policies upon diet identified by Jaime and Lock [11] and Van Cauwenberghe [14], were not found to extend to improved weight status in this review most likely due to the difficulties in accurately assessing diet. Nutrition guidelines formed a component in each of the combined policies which may indicate that diet related policies are beneficial when used in combination with physical activity policies. More evidence was found to support the introduction of physical activity policies to affect weight status with some evidence found to support the improvement of the quality and variety of PE identified by Lagarde and LeBlanc [15] which were also components in the AVHPSP [58], Be Active Eat Well [27,31], Gold Medal Schools [64] and Healthy Living Cambridge Kids [63]. However, results relating to professionally led PE and the duration and frequency of PE and break periods were mixed. Although there was a lack of significant findings for diet and physical activity policies by themselves (Figures 4 and 5) the overall result of the Be Active Eat Well programme (which encouraged the development of healthy eating and physical activity policies) was a significant reduction in BMI-SDS (Figure 6). This suggests that the process of policy development, engagement and broader activities may be more important than the presence or absence of a policy, supporting the need for policies to be implemented as part of a multifaceted intervention programme. The overall effect of each of the included multifaceted intervention programmes, was less than one BMI-SDS which is only equivalent to a change in weight of around 2.0 kg in Reception or 6.4 kg in Year 6 aged children. Rose and Day [67] have demonstrated that small changes in population mean values like those observed produce significant reductions in the prevalence of conditions like overweight and obesity. More recently, Kolsgaard, *et al.*[68] found significant physiological improvements (lower insulin and cholesterol) among obese children and adolescents from very small changes in BMI-SDS (<0.1). There has been discussion regarding shifting the focus from weight loss to improving health and fitness which may not require or result in weight loss through initiatives like Health At Every Size (HAES) as it is possible to be fit and fat [69]. Subsequently, further discussion is required upon what constitutes an important or clinically significant effect of obesity prevention or health promotion interventions.

## Conclusion

The evidence from this systematic review suggests that diet and physical activity related policies need to be located within more complex approaches to preventing childhood obesity which focus on multiple factors (e.g. diet, physical activity, sedentary behaviour, self-esteem) and at multiple levels of influence (e.g. home, school, neighbourhood) as advocated by the Centers for Disease Control and Prevention guidelines [10]. No policies which guided choice through disincentives, or eliminated choice were identified during the review, which may be pertinent as these policy actions have been effectively employed in campaigns to reduce the prevalence of smoking [7]. Although there are calls for similar policy actions to prevent further increases in the prevalence of obesity, the policy would need to extend outside of schools [6].

The complex web of factors which influence weight have been illustrated in the obesity systems map which also highlights the range of levels of influence from micro to macro [1]. Within this systematic review insufficient evidence was found to make recommendations upon the use of policies which aim to influence only one factor related to weight status (diet or physical activity) and at one level of influence (school). However, these results suggest that policies need to be located within wider health promotion intervention programmes in order to have an effect [10]. Further research is going to be crucial to the development and commissioning of evidence based policy and therefore, policy makers and researchers should work in partnership to consider the evaluation of new policies prior to implementation. Although there are difficulties in implementing new policies experimentally, such as blinding of outcome assessment and loss to follow-up, making use of the natural variation in uptake of policies to research the effects on weight status, so-called natural experiments (e.g. controlled before and after studies, interrupted time series studies) could be used to evaluate new policies [70,71]. The difficulties encountered in this review highlight the need for future studies to be comprehensively reported and have a duration of years rather than months, in order to inform future systematic reviews and meta-analyses.

## Abbreviations

95% CI: 95% confidence interval; AVHPSP: Annapolis valley health promoting schools program; BMI: Body mass index; BMI%: Body mass index percentile; BMIHFZ: Body mass index healthy fitness zone; BMI-SDS: Body mass index standard deviation score; CDC: Centers for disease control and prevention; ECLS-K: Early childhood longitudinal study – kindergarten cohort; FMI: Fat mass index; HAES: Health at every size; ICC: Intra-cluster correlation; IOTF: International obesity task force; MeSH: Medical subject headings; NSLP: National school lunch program; PE: Physical education; SBP: School breakfast program; UK: United Kingdom; USA: United States of America; USDA: United States Department of Agriculture.

## Competing interest

The authors declare that they have no competing interests.

## Authors’ contributions

AJW was involved with the conception and design of the review, undertook the searches and participated in the study identification, data extraction and quality assessment, he then undertook the analysis and drafted the manuscript. WEH was involved with the conception and design of the review, advised on and supervised the analysis and assisted with drafting the manuscript. CAW was involved with the conception of the study and had input into the final manuscript. AJH participated in the study identification, data extraction and quality assessment and proofread the final manuscript. SL contributed to the drafting of the final manuscript and interpretation of the results. KMW was involved with the conception and design of the review, participated in the data extraction and quality assessment, assisted with the interpretation of results and drafting of the final manuscript. All authors read and approved the final manuscript.

## Supplementary Material

Additional file 1 Search strategy. Click here for file

Additional file 2 Sensitivity analysis. Click here for file

Additional file 3 Effect size calculations. Click here for file

Additional file 4 **Diet related policies meta-analysis with Rappaport, Daskalakis and Sendacki **[66]** replacing Foster, *et al. ***[55]**.** Click here for file
