# Supplementary material for: Systematic review and meta-analysis of the association between childhood overweight and obesity and primary school diet and physical activity policies
Source: Int J Behav Nutr Phys Act. 2013 Aug 22;10:101. doi: 10.1186/1479-5868-10-101 (PMC3844408; doi:10.1186/1479-5868-10-101)
Supplement: Additional file 1 — Search strategy. [file 1479-5868-10-101-S1.docx]

**Additional file 1** – Medline search strategy

***Diet related policies search strategy*** - (**A** AND (**B** AND **C**)) AND **E**

***Physical activity related policies search strategy* -** (**A** AND (**B** AND **D**)) AND **E**

| **#** | **Population search terms** |
| --- | --- |
| 1 | exp Education, Nonprofessional/ |
| 2 | exp Child/ |
| 3 | ((primary or junior or elementary) adj2 (school or schools)).ti,ab. |
| 4 | (primary adj2 (pupil or pupils or schoolchildren)).ti,ab. |
| 5 | (elementary adj2 (pupil or pupils or schoolchildren)).ti,ab. |
| 6 | (junior adj2 (pupil or pupils or schoolchildren)).ti,ab. |
| 7 | (school and (child or children or infant*)).ti,ab. |
| 8 | (pre-school* or preschool*).ti,ab. |
| 9 | girl.ti,ab. |
| 10 | girls.ti,ab. |
| 11 | boy.ti,ab. |
| 12 | boys.ti,ab. |
| 13 | kid.ti,ab. |
| 14 | kids.ti,ab. |
| 15 | preadolescent.ti,ab. |
| 16 | preadolescence.ti,ab. |
| 17 | prepubescent.ti,ab. |
| **A** | 1 or 2 or 3 or 4 or 5 or 6 or 7 or 8 or 9 or 10 or 11 or 12 or 13 or 14 or 15 or 16 or 17 |

| **#** | **Policy intervention search terms** |
| --- | --- |
| 1 | exp Public Policy/ |
| 2 | exp Government Regulation/ |
| 3 | exp guideline/ |
| 4 | exp Legislation as Topic/ |
| 5 | exp "Facility Regulation and Control"/ |
| 6 | exp Health Promotion/ |
| 7 | evaluation studies as topic/ or exp feasibility studies/ or exp pilot projects/ or exp program evaluation/ |
| 8 | (school* adj2 (program* or intervention* or polic* or situational factors)).ti,ab. |
| 9 | (government regulation).ti,ab. |
| 10 | guideline*.ti,ab. |
| 11 | legislation.ti,ab. |
| 12 | (public polic*).ti,ab. |
| 13 | (policy making).ti,ab. |
| 14 | (facility regulation).ti,ab. |
| 15 | (facility control).ti,ab. |
| 16 | (health promot*).ti,ab. |
| 17 | (health behaviour).ti,ab. |
| 18 | (evaluation stud*).ti,ab. |
| **B** | 1 or 2 or 3 or 4 or 5 or 6 or 7 or 8 or 9 or 10 or 11 or 12 or 13 or 14 or 15 or 16 or 17 or 18 |

| **#** | **Diet intervention search terms** |
| --- | --- |
| 1 | exp Vegetables/ |
| 2 | exp Fruit/ |
| 3 | exp Dietary Fats/ |
| 4 | exp Sodium Chloride, Dietary/ |
| 5 | exp Carbonated Beverages/ |
| 6 | exp Energy Intake/ |
| 7 | (school food environment).ti,ab. |
| 8 | (school nutrition).ti,ab. |
| 9 | (school food polic*).ti,ab. |
| 10 | (nutrition polic*).ti,ab. |
| 11 | (competitive food*).ti,ab. |
| 12 | vegetable*.ti,ab. |
| 13 | (vegetable intake).ti,ab. |
| 14 | (vegetable consumption).ti,ab. |
| 15 | fruit*.ti,ab. |
| 16 | (fruit intake).ti,ab. |
| 17 | (fruit consumption).ti,ab. |
| 18 | fat.ti,ab. |
| 19 | (fat intake).ti,ab. |
| 20 | (fat consumption).ti,ab. |
| 21 | salt.ti,ab. |
| 22 | (salt intake).ti,ab. |
| 23 | (salt consumption).ti,ab. |
| 24 | (soft drink).ti,ab. |
| 25 | (soft drink intake).ti,ab. |
| 26 | (soft drink consumption).ti,ab. |
| 27 | (sweetened beverage).ti,ab. |
| 28 | (sweetened beverage intake).ti,ab. |
| 29 | (sweetened beverage consumption).ti,ab. |
| 30 | (food choice*).ti,ab. |
| 31 | (five-a-day).ti,ab. |
| 32 | (child nutrition).ti,ab. |
| 33 | (healthy eating).ti,ab. |
| 34 | (energy intake).ti,ab. |
| 35 | (caloric intake).ti,ab. |
| 36 | (school lunch*).ti,ab. |
| 37 | (school meal*).ti,ab. |
| 38 | (menu plan*).ti,ab. |
| 39 | (feeding program*).ti,ab. |
| 40 | canteen.ti,ab. |
| 41 | cafeteria.ti,ab. |
| 42 | (food service*).ti,ab. |
| **C** | 1 or 2 or 3 or 4 or 5 or 6 or 7 or 8 or 9 or 10 or 11 or 12 or 13 or 14 or 15 or 16 or 17 or 18 or 19 or 20 or 21 or 22 or 23 or 24 or 25 or 26 or 27 or 28 or 29 or 30 or 31 or 32 or 33 or 34 or 35 or 36 or 37 or 38 or 39 or 40 or 41 or 42 |

| **#** | **Physical activity intervention search terms** |
| --- | --- |
| 1 | exp Recreation/ *includes sports* |
| 2 | (physical and (activit* or education)).ti,ab. |
| 3 | (enrichment or recreation or sport).ti,ab. |
| 4 | (school and (exercise* or fitness or fit or activit* or sport* or leisure) adj3 (program* or facilit* or centre* or center* or organi?ation* or regime* or scheme* or class* or strateg* or club* or service*)).ti,ab. |
| 5 | (play ground).ti,ab. |
| 6 | playground.ti,ab. |
| 7 | (playground mark*).ti,ab. |
| 8 | (playground facilit*).ti,ab. |
| 9 | (playing field).ti,ab. |
| **D** | 1 or 2 or 3 or 4 or 5 or 6 or 7 or 8 or 9 |

| **#** | **Outcome search terms** |
| --- | --- |
| 1 | body weight/ or body weight changes/ or weight gain/ or weight loss/ or overweight/ or obesity/ or obesity, morbid/ or thinness/ |
| 2 | body constitution/ or "body weights and measures"/ or body fat distribution/ or adiposity/ or body mass index/ or body size/ or body height/ or body weight/ or ideal body weight/ or overweight/ or obesity/ or obesity, abdominal/ or obesity, morbid/ or thinness/ or waist circumference/ or skinfold thickness/ or waist-hip ratio/ |
| 3 | obes*.ti,ab. |
| 4 | (over weight).ti,ab. |
| 5 | overweight.ti,ab. |
| 6 | BMI.ti,ab. |
| 7 | (body mass).ti,ab. |
| 8 | (body mass index).ti,ab. |
| 9 | (body fat).ti,ab. |
| 10 | (body composition).ti,ab. |
| 11 | (body weight).ti,ab. |
| 12 | (body shape).ti,ab. |
| 13 | (waist circumference).ti,ab. |
| 14 | skinfold.ti,ab. |
| 15 | (skin fold).ti,ab. |
| 16 | (waist to hip ratio).ti,ab. |
| 17 | (waist-hip ratio).ti,ab. |
| 18 | (waist to height ratio).ti,ab. |
| 19 | (waist-height ratio).ti,ab. |
| 20 | (abdominal fat).ti,ab. |
| 21 | adiposity.ti,ab. |
| 22 | IOTF.ti,ab. |
| 23 | (international obesity task force).ti,ab. |
| 24 | (international obesity taskforce).ti,ab. |
| 25 | (weight adj2 gain).ti,ab. |
| 26 | (weight adj2 loss).ti,ab. |
| 27 | (weight adj2 change).ti,ab. |
| 28 | (BMI adj2 gain).ti,ab. |
| 29 | (BMI adj2 loss).ti,ab. |
| 30 | (BMI adj2 change).ti,ab. |
| 31 | ((body mass index) adj2 gain).ti,ab. |
| 32 | ((body mass index) adj2 loss).ti,ab. |
| 33 | ((body mass index) adj2 change).ti,ab. |
| **E** | 1 or 2 or 3 or 4 or 5 or 6 or 7 or 8 or 9 or 10 or 11 or 12 or 13 or 14 or 15 or 16 or 17 or 18 or 19 or 20 or 21 or 22 or 23 or 24 or 25 or 26 or 27 or 28 or 29 or 30 or 31 or 32 or 33 |
