# Supplementary material for: Systematic review and meta-analysis of the association between childhood overweight and obesity and primary school diet and physical activity policies
Source: Int J Behav Nutr Phys Act. 2013 Aug 22;10:101. doi: 10.1186/1479-5868-10-101 (PMC3844408; doi:10.1186/1479-5868-10-101)
Supplement: Additional file 2 — Sensitivity analysis. [file 1479-5868-10-101-S2.docx]

**Additional file 2** – Sensitivity analysis

**National School Lunch Program** – random effects meta-analysis

|  |  |  | **Correlation between pre- and post- scores** | | | | | | | | | | |
| --- | --- | --- | --- | --- | --- | --- | --- | --- | --- | --- | --- | --- | --- |
|  |  |  | **r=0.4** | | |  | **r=0.6** | | |  | **r=0.8** | | |
|  |  | **Study** | **ES** | **95% CI** | **% Weight** |  | **ES** | **95% CI** | **% Weight** |  | **ES** | **95% CI** | **% Weight** |
| **Covariate outcome correlation or multiple correlation** | **r=0.1** | Henry, 2006 [50] | 1.393 | 0.549, 2.237 | 6.62 |  | 1.393 | 0.549, 2.237 | 6.62 |  | 1.393 | 0.549, 2.237 | 6.62 |
|  |  | Hernandez. Francis and Doyle, 2011 [41]* | 0.017 | -0.154, 0.188 | 31.07 |  | 0.017 | -0.154, 0.188 | 31.07 |  | 0.017 | -0.154, 0.188 | 31.07 |
|  |  | Jones, *et al.* 2003 [34] | -0.325 | -0.585, -0.064 | 25.73 |  | -0.325 | -0.585, -0.064 | 25.73 |  | -0.325 | -0.585, -0.064 | 25.73 |
|  |  | Millimet, Tchernis and Husain, 2008 [43] and 2010 [44]* | 0.052 | 0.014, 0.091 | 36.58 |  | 0.052 | 0.014, 0.091 | 36.58 |  | 0.052 | 0.014, 0.091 | 36.58 |
|  |  | **D+L pooled ES** | 0.033 | -0.207, 0.273 | 100.00 |  | 0.033 | -0.207, 0.273 | 100.00 |  | 0.033 | -0.207, 0.273 | 100.00 |
|  |  | **Heterogeneity** | I^2^ = 83.1%, p<0.001 | |  |  | I^2^ = 83.1%, p<0.001 | |  |  | I^2^ = 80.8%, p=0.001 | |  |
|  |  |  |  |  |  |  |  |  |  |  |  |  |  |
|  | **r=0.3** | Henry, 2006 [50] | 1.393 | 0.549, 2.237 | 6.27 |  | 1.393 | 0.549, 2.237 | 6.27 |  | 1.393 | 0.549, 2.237 | 6.27 |
|  |  | Hernandez. Francis and Doyle, 2011 [41]* | 0.016 | -0.148, 0.181 | 32.41 |  | 0.016 | -0.148, 0.181 | 32.41 |  | 0.016 | -0.148, 0.181 | 32.41 |
|  |  | Jones, *et al.* 2003 [34] | -0.325 | -0.631, -0.018 | 23.02 |  | -0.325 | -0.631, -0.018 | 23.02 |  | -0.325 | -0.631, -0.018 | 23.02 |
|  |  | Millimet, Tchernis and Husain, 2008 [43] and 2010 [44]* | 0.052 | 0.010, 0.094 | 38.29 |  | 0.052 | 0.010, 0.094 | 38.29 |  | 0.052 | 0.010, 0.094 | 38.29 |
|  |  | **D+L pooled ES** | 0.038 | -0.193, 0.269 | 100.00 |  | 0.038 | -0.193, 0.269 | 100.00 |  | 0.038 | -0.193, 0.269 | 100.00 |
|  |  | **Heterogeneity** | I^2^ = 80.8%, p=0.001 | |  |  | I^2^ = 80.8%, p=0.001 | |  |  | I^2^ = 80.8%, p=0.001 | |  |
|  |  |  |  |  |  |  |  |  |  |  |  |  |  |
|  | **r=0.5** | Henry, 2006 [50] | 1.393 | 0.549, 2.237 | 5.65 |  | 1.393 | 0.549, 2.237 | 5.65 |  | 1.393 | 0.549, 2.237 | 5.65 |
|  |  | Hernandez. Francis and Doyle, 2011 [41]* | 0.015 | -0.135, 0.164 | 34.37 |  | 0.015 | -0.135, 0.164 | 34.37 |  | 0.015 | -0.135, 0.164 | 34.37 |
|  |  | Jones, *et al.* 2003 [34] | -0.325 | -0.678, -0.029 | 19.58 |  | -0.325 | -0.678, -0.029 | 19.58 |  | -0.325 | -0.678, -0.029 | 19.58 |
|  |  | Millimet, Tchernis and Husain, 2008 [43] and 2010 [44]* | 0.052 | 0.007, 0.098 | 40.41 |  | 0.052 | 0.007, 0.098 | 40.41 |  | 0.052 | 0.007, 0.098 | 40.41 |
|  |  | **D+L pooled ES** | 0.041 | -0.175, 0.257 | 100.00 |  | 0.041 | -0.175, 0.257 | 100.00 |  | 0.041 | -0.175, 0.257 | 100.00 |
|  |  | **Heterogeneity** | I^2^ = 79.0%, p=0.003 | |  |  | I^2^ = 79.0%, p=0.003 | |  |  | I^2^ = 79.0%, p=0.003 | |  |

*Study using the Early Childhood Longitudinal Study – Kindergarten (ECLS-K) cohort

95% CI; 95% confidence interval, ES; effect size, D+L; DerSimonian and Laird

**School Breakfast Program** – random effects meta-analysis

|  |  |  | **Correlation between pre- and post- scores** | | | | | | | | | | |
| --- | --- | --- | --- | --- | --- | --- | --- | --- | --- | --- | --- | --- | --- |
|  |  |  | **r=0.4** | | |  | **r=0.6** | | |  | **r=0.8** | | |
|  |  | **Study** | **ES** | **95% CI** | **% Weight** |  | **ES** | **95% CI** | **% Weight** |  | **ES** | **95% CI** | **% Weight** |
| **Covariate outcome correlation or multiple correlation** | **r=0.1** | Baxter, *et al.* 2009 [53] | -0.113 | -0.230, 0.003 | 16.78 |  | -0.113 | -0.230, 0.003 | 16.78 |  | -0.113 | -0.230, 0.003 | 16.77 |
|  |  | Jones, *et al.* 2003 [34] | -0.159 | -0.364, 0.046 | 7.64 |  | -0.159 | -0.364, 0.046 | 7.65 |  | -0.159 | -0.364, 0.046 | 7.64 |
|  |  | Millimet and Tchernis, 2009 [42]* | -0.116 | -0.139, -0.094 | 37.23 |  | -0.116 | -0.139, -0.094 | 37.15 |  | -0.116 | -0.139, -0.094 | 37.10 |
|  |  | Millimet, Tchernis and Husain, 2008 [43] and 2010 [44]* | -0.029 | -0.067, 0.010 | 34.10 |  | -0.029 | -0.067, 0.010 | 34.04 |  | -0.029 | -0.067, 0.010 | 34.00 |
|  |  | Ramirez-Lopez, *et al.* 2005 [54] | 0.034 | -0.255, 0.323 | 4.26 |  | 0.037 | -0.248, 0.322 | 4.38 |  | 0.037 | -0.244, 0.318 | 4.49 |
|  |  | **D+L pooled ES** | -0.083 | -0.146, -0.020 | 100.00 |  | -0.082 | -0.146, -0.019 | 100.00 |  | -0.082 | -0.145, -0.019 | 100.00 |
|  |  | **Heterogeneity** | I^2^ = 75.0%, p=0.003 | |  |  | I^2^ = 75.1%, p=0.003 | |  |  | I^2^ = 75.2%, p=0.003 | |  |
|  |  |  |  |  |  |  |  |  |  |  |  |  |  |
|  | **r=0.3** | Baxter, *et al.* 2009 [53] | -0.113 | -0.230, 0.003 | 16.85 |  | -0.113 | -0.230, 0.003 | 16.84 |  | -0.113 | -0.230, 0.003 | 16.83 |
|  |  | Jones, *et al.* 2003 [34] | -0.159 | -0.400, 0.082 | 5.78 |  | -0.159 | -0.400, 0.082 | 5.78 |  | -0.159 | -0.400, 0.082 | 5.78 |
|  |  | Millimet and Tchernis, 2009 [42]* | -0.116 | -0.141, -0.092 | 38.01 |  | -0.116 | -0.141, -0.092 | 37.94 |  | -0.116 | -0.141, -0.092 | 37.85 |
|  |  | Millimet, Tchernis and Husain, 2008 [43] and 2010 [44]* | -0.029 | -0.070, 0.013 | 34.19 |  | -0.029 | -0.070, 0.013 | 34.13 |  | -0.029 | -0.070, 0.013 | 34.07 |
|  |  | Ramirez-Lopez, *et al.* 2005 [54] | 0.037 | -0.220, 0.294 | 5.17 |  | 0.037 | -0.216, 0.290 | 5.31 |  | 0.037 | -0.212, 0.286 | 5.47 |
|  |  | **D+L pooled ES** | -0.080 | -0.143, -0.018 | 100.00 |  | -0.080 | -0.143, -0.017 | 100.00 |  | -0.080 | -0.143, -0.017 | 100.00 |
|  |  | **Heterogeneity** | I^2^ = 71.2%, p=0.008 | |  |  | I^2^ = 71.3%, p=0.007 | |  |  | I^2^ = 71.4%, p=0.007 | |  |
|  |  |  |  |  |  |  |  |  |  |  |  |  |  |
|  | **r=0.5** | Baxter, *et al.* 2009 [53] | -0.113 | -0.230, 0.003 | 16.82 |  | -0.113 | -0.230, 0.003 | 16.81 |  | -0.113 | -0.230, 0.003 | 16.80 |
|  |  | Jones, *et al.* 2003 [34] | -0.159 | -0.436, 0.119 | 4.47 |  | -0.159 | -0.436, 0.119 | 4.48 |  | -0.159 | -0.436, 0.119 | 4.48 |
|  |  | Millimet and Tchernis, 2009 [42]* | -0.116 | -0.142, -0.090 | 38.20 |  | -0.116 | -0.142, -0.090 | 38.09 |  | -0.116 | -0.142, -0.090 | 37.97 |
|  |  | Millimet, Tchernis and Husain, 2008 [43] and 2010 [44]* | -0.029 | -0.074, 0.016 | 33.78 |  | -0.029 | -0.074, 0.016 | 33.70 |  | -0.029 | -0.074, 0.016 | 33.61 |
|  |  | Ramirez-Lopez, *et al.* 2005 [54] | 0.037 | -0.182, 0.256 | 6.72 |  | 0.037 | -0.179, 0.253 | 6.92 |  | 0.037 | -0.175, 0.248 | 7.14 |
|  |  | **D+L pooled ES** | -0.078 | -0.140, -0.016 | 100.00 |  | -0.077 | -0.140, -0.015 | 100.00 |  | -0.077 | -0.139, -0.015 | 100.00 |
|  |  | **Heterogeneity** | I^2^ = 68.1%, p=0.014 | |  |  | I^2^ = 68.3%, p=0.013 | |  |  | I^2^ = 68.4%, p=0.013 | |  |

*Study using the Early Childhood Longitudinal Study – Kindergarten (ECLS-K) cohort

95% CI; 95% confidence interval, ES; effect size, D+L; DerSimonian and Laird

**Other diet related policies** – random effects meta-analysis

|  |  |  | **Correlation between pre- and post- scores** | | | | | | | | | | | |
| --- | --- | --- | --- | --- | --- | --- | --- | --- | --- | --- | --- | --- | --- | --- |
|  |  |  | **r=0.4** | | | |  | **r=0.6** | | |  | **r=0.8** | | |
|  |  | **Study** | **ES** | **95% CI** | | **% Weight** |  | **ES** | **95% CI** | **% Weight** |  | **ES** | **95% CI** | **% Weight** |
| **Covariate outcome correlation or multiple correlation** | **r=0.1** | Foster, *et al.* 2008 [55] | -0.006 | -0.080, 0.068 | | 36.29 |  | -0.006 | -0.080, 0.068 | 36.29 |  | -0.006 | -0.080, 0.068 | 36.29 |
|  |  | Fox, *et al.* 2009 [56] | -0.027 | -0.123, 0.069 | | 21.42 |  | -0.027 | -0.123, 0.069 | 21.42 |  | -0.027 | -0.123, 0.069 | 21.42 |
|  |  | Johnson, *et al.* 2012 [31] | -0.040 | -0.133, 0.053 | | 22.95 |  | -0.040 | -0.133, 0.053 | 22.95 |  | -0.040 | -0.133, 0.053 | 22.95 |
|  |  | Veugelers and Fitzgerald, 2005 [58] | -0.026 | -0.127, 0.075 | | 19.33 |  | -0.026 | -0.127, 0.075 | 19.33 |  | -0.026 | -0.127, 0.075 | 19.33 |
|  |  | **D+L pooled ES** | -0.022 | -0.067, 0.022 | | 100.00 |  | -0.022 | -0.067, 0.022 | 100.00 |  | -0.022 | -0.067, 0.022 | 100.00 |
|  |  | **Heterogeneity** | I^2^ = 0.0%, p=0.952 | | |  |  | I^2^ = 0.0%, p=0.952 | |  |  | I^2^ = 0.0%, p=0.952 | |  |
|  |  |  |  | |  |  |  |  |  |  |  |  |  |  |
|  | **r=0.3** | Foster, *et al.* 2008 [55] | -0.006 | | -0.079, 0.067 | 37.77 |  | -0.006 | -0.079, 0.067 | 37.77 |  | -0.006 | -0.079, 0.067 | 37.77 |
|  |  | Fox, *et al.* 2009 [56] | -0.026 | | -0.127, 0.075 | 19.89 |  | -0.026 | -0.127, 0.075 | 19.89 |  | -0.026 | -0.127, 0.075 | 19.89 |
|  |  | Johnson, *et al.* 2012 [31] | -0.038 | | -0.127, 0.051 | 25.41 |  | -0.038 | -0.127, 0.051 | 25.41 |  | -0.038 | -0.127, 0.051 | 25.41 |
|  |  | Veugelers and Fitzgerald, 2005 [58] | -0.026 | | -0.135, 0.083 | 16.93 |  | -0.026 | -0.135, 0.083 | 16.93 |  | -0.026 | -0.135, 0.083 | 16.93 |
|  |  | **D+L pooled ES** | -0.021 | | -0.066, 0.023 | 100.00 |  | -0.021 | -0.066, 0.023 | 100.00 |  | -0.021 | -0.066, 0.023 | 100.00 |
|  |  | **Heterogeneity** | I^2^ = 0.0%, p=0.954 | | |  |  | I^2^ = 0.0%, p=0.954 | |  |  | I^2^ = 0.0%, p=0.954 | |  |
|  |  |  |  | |  |  |  |  |  |  |  |  |  |  |
|  | **r=0.5** | Foster, *et al.* 2008 [55] | -0.005 | | -0.076, 0.066 | 38.28 |  | -0.005 | -0.076, 0.066 | 38.28 |  | -0.005 | -0.076, 0.066 | 38.28 |
|  |  | Fox, *et al.* 2009 [56] | -0.024 | | -0.127, 0.080 | 17.99 |  | -0.024 | -0.127, 0.080 | 17.99 |  | -0.024 | -0.127, 0.080 | 17.99 |
|  |  | Johnson, *et al.* 2012 [31] | -0.035 | | -0.116, 0.046 | 29.50 |  | -0.035 | -0.116, 0.046 | 29.50 |  | -0.035 | -0.116, 0.046 | 29.50 |
|  |  | Veugelers and Fitzgerald, 2005 [58] | -0.026 | | -0.142, 0.090 | 14.22 |  | -0.026 | -0.142, 0.090 | 14.22 |  | -0.026 | -0.142, 0.090 | 14.22 |
|  |  | **D+L pooled ES** | -0.020 | | -0.064, 0.024 | 100.00 |  | -0.020 | -0.064, 0.024 | 100.00 |  | -0.020 | -0.064, 0.024 | 100.00 |
|  |  | **Heterogeneity** | I^2^ = 0.0%, p=0.958 | | |  |  | I^2^ = 0.0%, p=0.958 | |  |  | I^2^ = 0.0%, p=0.958 | |  |

95% CI; 95% confidence interval, ES; effect size, D+L; DerSimonian and Laird

**Physical activity related policies** – random effects meta-analysis

|  |  |  | **Correlation between pre- and post- scores** | | | | | | | | | | |
| --- | --- | --- | --- | --- | --- | --- | --- | --- | --- | --- | --- | --- | --- |
|  |  |  | **r=0.4** | | |  | **r=0.6** | | |  | **r=0.8** | | |
|  |  | **Study** | **ES** | **95% CI** | **% Weight** |  | **ES** | **95% CI** | **% Weight** |  | **ES** | **95% CI** | **% Weight** |
| **Covariate outcome correlation or multiple correlation** | **r=0.1** | Chiodera, *et al.* 2008 [60] | -0.010 | -0.049, 0.028 | 50.04 |  | -0.010 | -0.042, 0.021 | 60.04 |  | -0.010 | -0.033, 0.012 | 75.03 |
|  |  | Donnelly, *et al.* 2009 [62] | 0.011 | -0.091, 0.113 | 7.15 |  | 0.011 | -0.091, 0.113 | 5.72 |  | 0.011 | -0.091, 0.113 | 3.57 |
|  |  | Fernandes, 2010 [39] and Fernandes and Sturm, 2011 [40]* | -0.009 | -0.056, 0.038 | 33.44 |  | -0.009 | -0.056, 0.038 | 26.75 |  | -0.009 | -0.056, 0.038 | 16.17 |
|  |  | Heelan, *et al.* 2009 [61] | -0.071 | -0.316, 0.174 | 1.23 |  | -0.071 | -0.316, 0.174 | 0.99 |  | -0.071 | -0.316, 0.174 | 0.62 |
|  |  | Johnson, *et al.* 2012 [31] | -0.044 | -0.147, 0.059 | 6.99 |  | -0.044 | -0.147, 0.059 | 5.59 |  | -0.044 | -0.147, 0.059 | 3.49 |
|  |  | Zhu, *et al.* 2010 [59] | -0.012 | -0.266, 0.242 | 1.15 |  | -0.012 | -0.266, 0.242 | 0.92 |  | -0.012 | -0.266, 0.242 | 0.57 |
|  |  | **D+L pooled ES** | -0.011 | -0.039, 0.016 | 100.00 |  | -0.011 | -0.036, 0.013 | 100.00 |  | -0.011 | -0.030, 0.008 | 100.00 |
|  |  | **Heterogeneity** | I^2^ = 0.0%, p=0.975 | |  |  | I^2^ = 0.0%, p=0.975 | |  |  | I^2^ = 0.0%, p=0.975 | |  |
|  |  |  |  |  |  |  |  |  |  |  |  |  |  |
|  | **r=0.3** | Chiodera, *et al.* 2008 [60] | -0.010 | -0.049, 0.028 | 52.29 |  | -0.010 | -0.042, 0.021 | 62.18 |  | -0.010 | -0.033, 0.012 | 76.68 |
|  |  | Donnelly, *et al.* 2009 [62] | 0.011 | -0.091, 0.113 | 7.47 |  | 0.011 | -0.091, 0.113 | 5.92 |  | 0.011 | -0.091, 0.113 | 3.65 |
|  |  | Fernandes, 2010 [39] and Fernandes and Sturm, 2011 [40]* | -0.009 | -0.060, 0.042 | 29.84 |  | -0.009 | -0.060, 0.042 | 23.66 |  | -0.009 | -0.060, 0.042 | 14.59 |
|  |  | Heelan, *et al.* 2009 [61] | -0.071 | -0.320, 0.177 | 1.25 |  | -0.071 | -0.320, 0.177 | 0.99 |  | -0.071 | -0.320, 0.177 | 0.61 |
|  |  | Johnson, *et al.* 2012 [31] | -0.042 | -0.141, 0.056 | 7.95 |  | -0.042 | -0.141, 0.056 | 6.30 |  | -0.042 | -0.141, 0.056 | 3.88 |
|  |  | Zhu, *et al.* 2010 [59] | -0.012 | -0.266, 0.242 | 1.20 |  | -0.012 | -0.266, 0.242 | 0.95 |  | -0.012 | -0.266, 0.242 | 0.59 |
|  |  | **D+L pooled ES** | -0.012 | -0.039, 0.016 | 100.00 |  | -0.011 | -0.036, 0.013 | 100.00 |  | -0.011 | -0.030, 0.008 | 100.00 |
|  |  | **Heterogeneity** | I^2^ = 0.0%, p=0.977 | |  |  | I^2^ = 0.0%, p=0.977 | |  |  | I^2^ = 0.0%, p=0.977 | |  |
|  |  |  |  |  |  |  |  |  |  |  |  |  |  |
|  | **r=0.5** | Chiodera, *et al.* 2008 [60] | -0.010 | -0.049, 0.028 | 53.46 |  | -0.010 | -0.042, 0.021 | 63.27 |  | -0.010 | -0.033, 0.012 | 77.51 |
|  |  | Donnelly, *et al.* 2009 [62] | 0.011 | -0.091, 0.113 | 7.64 |  | 0.011 | -0.091, 0.113 | 6.03 |  | 0.011 | -0.091, 0.113 | 3.69 |
|  |  | Fernandes, 2010 [39] and Fernandes and Sturm, 2011 [40]* | -0.009 | -0.063, 0.046 | 26.58 |  | -0.009 | -0.063, 0.046 | 20.97 |  | -0.009 | -0.063, 0.046 | 12.84 |
|  |  | Heelan, *et al.* 2009 [61] | -0.071 | -0.323, 0.180 | 1.25 |  | -0.071 | -0.323, 0.180 | 0.98 |  | -0.071 | -0.323, 0.180 | 0.60 |
|  |  | Johnson, *et al.* 2012 [31] | -0.038 | -0.128, 0.051 | 9.86 |  | -0.038 | -0.128, 0.051 | 7.78 |  | -0.038 | -0.128, 0.051 | 4.76 |
|  |  | Zhu, *et al.* 2010 [59] | -0.012 | -0.266, 0.242 | 1.22 |  | -0.012 | -0.266, 0.242 | 0.97 |  | -0.012 | -0.266, 0.242 | 0.59 |
|  |  | **D+L pooled ES** | -0.012 | -0.040, 0.016 | 100.00 |  | -0.012 | -0.037, 0.013 | 100.00 |  | -0.011 | -0.031, 0.008 | 100.00 |
|  |  | **Heterogeneity** | I^2^ = 0.0%, p=0.979 | |  |  | I^2^ = 0.0%, p=0.979 | |  |  | I^2^ = 0.0%, p=0.979 | |  |

*Study using the Early Childhood Longitudinal Study – Kindergarten (ECLS-K) cohort

95% CI; 95% confidence interval, ES; effect size, D+L; DerSimonian and Laird

**Combined policies** – Individual Hedges’ g

|  |  |  | **Correlation between pre- and post- scores** | | | | | | | |
| --- | --- | --- | --- | --- | --- | --- | --- | --- | --- | --- |
|  |  |  | **r=0.4** | |  | **r=0.6** | |  | **r=0.8** | |
|  |  | **Study** | **ES** | **95% CI** |  | **ES** | **95% CI** |  | **ES** | **95% CI** |
| **Covariate outcome correlation or multiple correlation** | **r=0.1** | Chomitz, *et al.* 2010 [63] | -0.013 | -0.085, 0.060 |  | -0.013 | -0.084, 0.058 |  | -0.013 | -0.082, 0.057 |
|  |  | Johnson, *et al.* 2012 [31] | -0.096 | -0.188, -0.004 |  | -0.096 | -0.188, -0.004 |  | -0.096 | -0.188, -0.004 |
|  |  | Jordan, *et al.* 2008 [64] | -0.746 | -1.029, -0.463 |  | -0.746 | -1.029, -0.463 |  | -0.746 | -1.029, -0.463 |
|  |  | Veugelers and Fitzgerald, 2005 [58] | -0.246 | -0.461, -0.031 |  | -0.246 | -0.461, -0.031 |  | -0.246 | -0.461, -0.031 |
|  |  | Zhu, *et al.* 2010 [59] | <-0.001 | -0.446, 0.445 |  | <-0.001 | -0.446, 0.445 |  | <-0.001 | -0.446, 0.445 |
|  |  |  |  |  |  |  |  |  |  |  |
|  | **r=0.3** | Chomitz, *et al.* 2010 [63] | -0.013 | -0.085, 0.060 |  | -0.013 | -0.084, 0.058 |  | -0.013 | -0.082, 0.057 |
|  |  | Johnson, *et al.* 2012 [31] | -0.092 | -0.180, -0.004 |  | -0.092 | -0.180, -0.004 |  | -0.092 | -0.180, -0.004 |
|  |  | Jordan, *et al.* 2008 [64] | -0.746 | -1.029, -0.463 |  | -0.746 | -1.029, -0.463 |  | -0.746 | -1.029, -0.463 |
|  |  | Veugelers and Fitzgerald, 2005 [58] | -0.246 | -0.473, -0.018 |  | -0.246 | -0.473, -0.018 |  | -0.246 | -0.473, -0.018 |
|  |  | Zhu, *et al.* 2010 [59] | <-0.001 | -0.468, 0.468 |  | <-0.001 | -0.468, 0.468 |  | <-0.001 | -0.468, 0.468 |
|  |  |  |  |  |  |  |  |  |  |  |
|  | **r=0.5** | Chomitz, *et al.* 2010 [63] | -0.013 | -0.086, 0.060 |  | -0.013 | -0.084, 0.059 |  | -0.013 | -0.082, 0.057 |
|  |  | Johnson, *et al.* 2012 [31] | -0.084 | -0.164, -0.004 |  | -0.084 | -0.164, -0.004 |  | -0.084 | -0.164, -0.004 |
|  |  | Jordan, *et al.* 2008 [64] | -0.746 | -1.029, -0.463 |  | -0.746 | -1.029, -0.463 |  | -0.746 | -1.029, -0.463 |
|  |  | Veugelers and Fitzgerald, 2005 [58] | -0.246 | -0.485, -0.006 |  | -0.246 | -0.485, -0.006 |  | -0.246 | -0.485, -0.006 |
|  |  | Zhu, *et al.* 2010 [59] | <-0.001 | -0.490, 0.489 |  | <-0.001 | -0.490, 0.489 |  | <-0.001 | -0.490, 0.489 |

95% CI; 95% confidence interval, ES; effect size
