# Supplementary material for: Systematic review and meta-analysis of the association between childhood overweight and obesity and primary school diet and physical activity policies
Source: Int J Behav Nutr Phys Act. 2013 Aug 22;10:101. doi: 10.1186/1479-5868-10-101 (PMC3844408; doi:10.1186/1479-5868-10-101)
Supplement: Additional file 3 — Effect size calculations. [file 1479-5868-10-101-S3.docx]

**Additional file 3** – Effect size calculations

| **Study** | **Reported outcome** | **Cohen’s *d* calculation** | **Notes** | **r** | **DF** | **-** | **C** |
| --- | --- | --- | --- | --- | --- | --- | --- |
| Baxter, *et al.* 2009 *[53]*[1] | BMI%: p-value of unadjusted coefficient | R: p_to_d2 | P-value of the difference in BMI% between those eating breakfast in the cafeteria and those eating it in the classroom. The number of students eating breakfast in the classroom or cafeteria was estimated based upon there being an equal number of students in each school and the data provided on the number of schools which used cafeteria or classroom. |  | ✓ | ✓ |  |
| Chiodera, *et al.* 2008 *[60]*[2] | BMI: pre- and post- policy means and standard deviations | Textbook: Computing *d* and *g* from studies that use pre-post scores or matched groups (p. 28) | Table 2 p.182: Using the data for both boys and girls combined (All) individual Cohen’s *d* were calculated for each grade prior to combination using Textbook: Computing a combined effect across outcomes (p.227). | ✓ | ✓ |  | ✓ |
| Chomitz, *et al.* 2010 *[63]*[3] | BMI-SDS: pre- and post- policy means and standard deviations | Textbook: Computing *d* and *g* from studies that use pre-post scores or matched groups (p. 28) | Table 2 p.S49  BMI-SDS Cohen’s *d* combined with overweight and obesity *d*-values using Textbook: Computing a combined effect across outcomes (p.227). | ✓ | ✓ |  | ✓ |
|  | Overweight and obesity: pre- and post- policy prevalence | Calculated odd ratio before R: lor_to_d | Table 3 p.S50: Using the total sample individual Cohen’s *d* were calculated for overweight and obesity. |  |  |  |  |
| Datar and Sturm, 2004 *[38]*[4] | BMI: adjusted regression coefficient with 95% confidence interval | Unable to calculate | Insufficient information on the number of participants in each exposure group prevented the calculation of an effect size, the corresponding author was contacted for this information but did not respond. |  |  |  |  |
| Donnelly, *et al.* 2009 *[62]*[5] | BMI: p-value of the difference in mean changes in BMI between the controls and those receiving the policy | R: p_to_d2 | Table 2 p.338 |  |  |  |  |
| Fernandes, 2010 [*39]*[6] and Fernandes and Sturm, 2011 *[40]*[7] | BMI%: adjusted regression coefficient with standard error | R: mean_to_d2 | School duration of PE and Recess meets the NASPE recommendations. The number of participants attending a school which met the recommendations were calculated for each grade using data from Table 2 [7] assuming that there were an equal number of students in each grade. Subsequently a Cohen’s *d*-value was calculated for each grade using the pre-BMI% from Table 2 [7] and calculating the post-BMI% as the pre-BMI% minus the regression coefficient from Table 3 [7]: overall girls and boys and all weight status. Each grade’s *d*-value was then combined using Textbook: Computing a combined effect across outcomes (p.227). |  |  |  | ✓ |
| Foster, *et al.* 2008 *[55]*[8]  and  Rappaport, Daskalakis and Sendacki *[66]*[9] | BMI-SDS: p-value of the adjusted difference between intervention and control | R: p.ancova_to_d2 | Table 3 p.e800  BMI-SDS Cohen’s *d* combined with overweight and obesity *d*-values using Textbook: Computing a combined effect across outcomes (p.227). | ✓ |  | ✓ | ✓ |
|  | Overweight and obesity prevalence: adjusted odds with 95% confidence interval | R: lor_to_d | Table 2 p.e798: The 95% confidence interval around the adjusted odds had to be back transformed into variance. |  |  |  |  |
| Fox, *et al.* 2009 *[56]*[10] | BMI-SDS: adjusted regression coefficient with standard error | R: p.ancova_to_d2 | Table 3 p.S115 Elementary school students: For each of the five policy statements the size of the exposed and non-exposed groups were calculated from the data in Table 1 p.S111. Then the coefficients were divided by the standard errors to give z-scores which were transformed into p-values. | ✓ |  | ✓ | ✓ |
|  | Obesity: adjusted odds ratio with 95% confidence interval | R: lor_to_d | Table 3 p.S115 Elementary school students: The 95% confidence interval around the adjusted odds had to be back transformed into variance. The *d*-values related to unhealthy foods were reversed so that they reflected the effect of removing rather than introducing the food. The *d*-value for BMI-SDS and obesity were combined for each policy statement and then all the policy statement *d*-values were combined using Textbook: Computing a combined effect across outcomes (p.227). |  |  |  |  |
| Harrison, *et al.* 2011 *[57]*[11] | - | Unable to calculate | Results presented symbolically rather than numerically. |  |  |  |  |
| Heelan, *et al.* 2009 *[61]*[12] | BMI-SDS: means and standard deviations | R: mean_to_d | Table 2 p.564: Cohen’s *d*-values were calculated for the three pairs: intervention and control, frequent walkers and passive commuters, and infrequent walkers and passive commuters. These *d*-values were then combined using Textbook: Computing a combined effect across outcomes (p.227). |  | ✓ |  | ✓ |
| Henry, 2006 *[50]*[13] | Overweight: constant standardised regression coefficient and standard error | Textbook: Converting from *r* to *d* (p. 48) | Table 7 p.73 Students on free- or reduced lunch program: the standardised coefficient was considered to be a *r*-value. Variance of the Cohen’s *d*-value calculated by firstly calculating the variance of *r* using Textbook: Computing *r* p.41, then converting this into *d*-value variance using Textbook: Converting from *r* to *d* p.48. |  |  |  |  |
| Hernandez. Francis and Doyle, 2011 *[41]*[14] | BMI: adjusted regression coefficients with standard errors | R: p.ancova_to_d2 | Table 3 p.351 Full sample: for each grade apart from eight the coefficient of ‘Participation in NSLP’ was divided by its standard error to give a z-score from which p-values were calculated enabling calculation of Cohen’s *d*-values. The Kindergarten, first, third and fifth grade *d*-values were combined using Textbook: Computing a combined effect across outcomes (p.227). | ✓ | ✓ |  | ✓ |
| Hinrichs, 2010 *[47]*[15] | BMI: adjusted regression coefficient with standard error | Did not calculate | This study examined adults who were exposed to a policy as children and therefore it was felt inappropriate to combine with the other studies. |  |  |  |  |
| Johnson, *et al.* 2012 *[31]*[16] | BMI-SDS: p-value of the adjusted difference between intervention and control | R: p.ancova_to_d2 | Table 2 p.904 Model 3b (individual+school): for each of the policies the size of the exposed and non-exposed groups were calculated from the data in Table 1 p.903 assuming equal numbers of students in each school. The sample sizes used were the mean of the intervention and control sample sizes. | ✓ |  | ✓ |  |
| Jones, *et al.* 2003 *[34]*[17] | Overweight or obese: adjusted odds ratio with 95% confidence interval | R: lor_to_d | Table 3 p.783: separate *d*-values were calculated for food secure and insecure boys and girls. The 95% confidence intervals were back transformed into variances. The *d*-values for ‘School lunch’ and ‘Both lunch and breakfast’ were calculated by combining the food secure and insecure boys and girls using Textbook: Computing a combined effect across outcomes (p.227). This study required design factor adjustment as it did not account for school clustering, however, there was insufficient information to enable the calculation of the average size of the cluster, therefore the standard errors may be larger than those reported [18]. |  | ? |  | ✓ |
| Jordan, *et al.* 2008 *[64]*[19] | BMI-SDS: means and standard errors | R: mean_to_d | Results and discussion paragraph 3 p.1917-8: without additional information it was assumed that there were an equal number of students in the intervention and control arms. |  |  |  |  |
| Millimet and Tchernis, 2009 *[42]*[20] | BMI growth rate: adjusted regression coefficient with 90% confidence interval | R: mean_to_d2 | Table 4 p.39 ATE $\tau_{MB-BC, 0.25}$ Specification 3: unexposed mean BMI growth rates taken from Table 3 p.38 and exposed BMI growth rates were calculated as the unexposed growth rates plus the regression coefficient. |  |  |  | ✓ |
|  | Overweight and obesity: adjusted regression coefficient with 90% confidence interval | R: lor_to_d | Table 5 and 6 p.40-1 ATE $\tau_{MB-BC, 0.25}$ Specification 3: odds ratios and their variances were calculated from the regression coefficients and data from Table 3 p.38. Separate *d*-values were calculated for third and fifth grade by combining the BMI growth rate, overweight and obesity Cohen’s *d*-values prior to combining third and fifth grade *d*-values all using Textbook: Computing a combined effect across outcomes (p.227). |  |  |  |  |
| Millimet, Tchernis and Husain, 2008 and 2010 *[43, 44]*[21, 22] | Overweight and obese: adjusted regression coefficient with standard errors | R: lor_to_d | Table 2 p.648 Full sample ρ=0.1 [22]: odds ratios and their variances were calculated from the regression coefficients and data from ‘Data’ paragraph 5 p.643-4. Separate *d*-values were calculated for ‘School lunch’ and ‘School breakfast’ by combining the overweight and obesity Cohen’s *d*-values using Textbook: Computing a combined effect across outcomes (p.227). |  |  |  | ✓ |
| Ramirez-Lopez, et al. 2005 *[54]*[23] | BMI: means and stand errors for pre- and post- SBP and control groups | Textbook: Computing *d* and *g* from studies that use pre-post scores or matched groups (p. 28) | Table 2: individual Cohen’s *d*-values were calculated for the SBP and control groups, these were then compared to give the overall *d*-values using Textbook: Comparing outcomes or time-points within a study (p. 233). | ✓ | ✓ |  | ✓ |
|  | Overweight or obese and obese: pre- and post- SBP and control groups prevalences | Calculated odd ratio before R: lor_to_d | Table 4: individual odds ratios were calculated for the SBP and control groups prior to conversion into Cohen’s *d*-values. The *d*-values were then compared to give the overall *d*-values using Textbook: Comparing outcomes or time-points within a study (p. 233). BMI, overweight or obese, and obese *d*-values were then combined using Textbook: Computing a combined effect across outcomes (p.227). |  |  |  |  |
| Veugelers and Fitzgerald, 2005 *[58]*[24] | Overweight and obese: adjusted odds ratio with 95% confidence interval | R: lor_to_d | Table 2 p.434: the 95% confidence intervals were back transformed into variances. Separate Cohen’s *d*-values were calculated for ‘Nutrition Program’ and ‘AVHPSP’ by combining the overweight and obese d-values using Textbook: Computing a combined effect across outcomes (p.227). |  |  |  | ✓ |
| Zhu, *et al.* 2010 *[59]*[25] | BMIHFZ: adjusted regression coefficient with standard errors | R: lor_to_d | Table 5 p.S59 Elementary school: odds ratios were calculated for each policy statement (Teacher training: education, PE/recess duration: days; length; Recess periods; Recess length, PE policy: Cancel due to weather; USDA; Wellness council; Exemptions from PE) using the intercept as the unexposed proportion and the intercept plus the coefficient as the exposed proportion. The odds ratios were converted into Cohen’s *d*-values and then the following policy statements were combined using Textbook: Computing a combined effect across outcomes (p.227), Physical activity policies: Teacher training: education, PE/recess duration: days; length; Recess periods; Recess length, PE policy: Cancel due to weather; Exemptions from PE, and Combined policies: USDA and Wellness council. |  |  | ✓ | ✓ |

-; result inverted in favour of policy intervention to reduce weight, AVHPSP; Annapolis Valley Health Promoting Schools Project, BMI; body mass index, BMI%; BMI percentile, BMIHFZ; BMI healthy fitness zone [26], BMI-SDS; BMI standard deviation score, C; results were combined from multiple outcomes or policy components using Textbook: Computing a combined effect across outcomes (p.227), DF; Design factor adjustment [18], NASPE; National Association of Sport and Physical Education, NSLP; National School Lunch Program, PE: physical education r; Multiple correlation assumption, R: MAd: Meta-Analysis with Mean Differences [27, 28], SBP; School Breakfast Program, Texbook: Introduction to meta-analysis [29], USDA; United States Department of Agriculture wellness program.

**References**

Reference number in italics are those from the manuscript.

1. Baxter SD, Royer JA, Hardin JW, Guinn CH, Mackelprang AJ: **Daily participation in school meals and 4th-grade children's age/sex body mass index percentile.** *FASEB J* 2009, **23**.

2. Chiodera P, Volta E, Gobbi G, Milioli MA, Mirandola P, Bonetti A, Delsignore R, Bernasconi S, Anedda A, Vitale M: **Specifically designed physical exercise programs improve children's motor abilities.** *Scand J Med Sci Sports* 2008, **18:**179-187.

3. Chomitz VR, McGowan RJ, Wendel JM, Williams SA, Cabral HJ, King SE, Olcott DB, Cappello M, Breen S, Hacker KA: **Healthy Living Cambridge Kids: a community-based participatory effort to promote healthy weight and fitness.** *Obesity (Silver Spring)* 2010, **18 Suppl 1:**S45-53.

4. Datar A, Sturm R: **Physical education in elementary school and body mass index: evidence from the early childhood longitudinal study.** *Am J Public Health* 2004, **94:**1501-1506.

5. Donnelly JE, Greene JL, Gibson CA, Smith BK, Washburn RA, Sullivan DK, DuBose K, Mayo MS, Schmelzle KH, Ryan JJ, et al: **Physical Activity Across the Curriculum (PAAC): a randomized controlled trial to promote physical activity and diminish overweight and obesity in elementary school children.** *Prev Med* 2009, **49:**336-341.

6. Fernandes MM: **Evaluating the impacts of school nutrition and physical activity policies on child health. PRGS Dissertation.** *Doctoral.* RAND Graduate School, 2010.

7. Fernandes MM, Sturm R: **The role of school physical activity programs in child body mass trajectory.** *J Phys Act Health* 2011, **8:**174-181.

8. Foster GD, Sherman S, Borradaile KE, Grundy KM, Vander Veur SS, Nachmani J, Karpyn A, Kumanyika S, Shults J: **A policy-based school intervention to prevent overweight and obesity.** *Pediatrics* 2008, **121:**e794-802.

9. Rappaport EB, Daskalakis C, Sendecki JA: **Using routinely collected growth data to assess a school-based obesity prevention strategy.** *Int J Obes (Lond)* 2013, **37:**79-85.

10. Fox MK, Dodd AH, Wilson A, Gleason PM: **Association between school food environment and practices and body mass index of US public school children.** *J Am Diet Assoc* 2009, **109:**S108-117.

11. Harrison F, Bentham G, Jones AP, Cassidy A, van Sluijs EM, Griffin SJ: **School level correlates with adiposity in 9-10 year old children.** *Health Place* 2011, **17:**710-716.

12. Heelan KA, Abbey BM, Donnelly JE, Mayo MS, Welk GJ: **Evaluation of a walking school bus for promoting physical activity in youth.** *J Phys Act Health* 2009, **6:**560-567.

13. Henry LL: **The influence of elementary-school programs on childhood obesity.** George Mason University, 2006.

14. Hernandez DC, Francis LA, Doyle EA: **National School Lunch Program participation and sex differences in body mass index trajectories of children from low-income families.** *Arch Pediatr Adolesc Med* 2011, **165:**346-353.

15. Hinrichs P: **The effects of the National School Lunch Program on education and health.** *J Policy Anal Manage* 2010, **29:**479-505.

16. Johnson BA, Kremer PJ, Swinburn BA, de Silva-Sanigorski AM: **Multilevel analysis of the Be Active Eat Well intervention: environmental and behavioural influences on reductions in child obesity risk.** *Int J Obes (Lond)* 2012, **36:**901-907.

17. Jones SJ, Jahns L, Laraia BA, Haughton B: **Lower risk of overweight in school-aged food insecure girls who participate in food assistance: results from the panel study of income dynamics child development supplement.** *Arch Pediatr Adolesc Med* 2003, **157:**780-784.

18. Higgins JPT, Green S (Eds.): **Cochrane handbook for systematic reviews of interventions. Version 5.1.0 [updated March 2011]**: The Cochrane Collaboration; 2011.

19. Jordan KC, Erickson ED, Cox R, Carlson EC, Heap E, Friedrichs M, Moyer-Mileur LJ, Shen S, Mihalopoulos NL: **Evaluation of the Gold Medal Schools program.** *J Am Diet Assoc* 2008, **108:**1916-1920.

20. Millimet DL, Tchernis R: **Estimation of treatment effects without an exclusion restriction: with an application to the analysis of the School Breakfast Program. NBER Working Paper No. 15539.** 2009.

21. Millimet DL, Tchernis R, Husain M: **School nutrition programs and the incidence of childhood obesity. NBER Working Paper No. 14297.** 2008.

22. Millimet DL, Tchernis R, Husain M: **School nutrition programs and the incidence of childhood obesity.** *J Hum Resour* 2010, **45:**640-654.

23. Ramirez-Lopez E, Grijalva-Haro MI, Valencia ME, Ponce JA, Artalejo E: **Effect of a School Breakfast Program on the prevalence of obesity and cardiovascular risk factors in children. [Spanish].** *Salud Publica Mex* 2005, **47:**126-133.

24. Veugelers PJ, Fitzgerald AL: **Effectiveness of school programs in preventing childhood obesity: a multilevel comparison.** *Am J Public Health* 2005, **95:**432-435.

25. Zhu W, Boiarskaia EA, Welk GJ, Meredith MD: **Physical education and school contextual factors relating to students' achievement and cross-grade differences in aerobic fitness and obesity.** *Res Q Exerc Sport* 2010, **81:**S53-64.

26. Going SB, Lohman TG, Falls HB: *Body composition assessment.* Dallas. TX: The Cooper Institute; 2008 [http://www.cooperinstitute.org/pub/file.cfm?item_type=xm_file&id=662] (Accessed 26 February 2013).

27. Del Re AC, Hoyt WT (2010). **MAd: Meta-Analysis with Mean Differences**. *R package version 0.8*, URL http://CRAN.R-project.org/package=MAd.

28. R Development Core Team (2011). **R: A language and environment for statistical computing**. *R Foundation for Statistical Computing*, Vienna, Austria. ISBN 3-900051-07-0, URL http://www.R-project.org/.

29. Borenstein M, Hedges LV, Higgins JPT, Rothstein HR: *Introduction to meta-analysis.* Oxford: Wiley; 2009.
