# Supplementary material for: Systematic review and meta-analysis of the association between childhood overweight and obesity and primary school diet and physical activity policies
Source: Int J Behav Nutr Phys Act. 2013 Aug 22;10:101. doi: 10.1186/1479-5868-10-101 (PMC3844408; doi:10.1186/1479-5868-10-101)
Supplement: Additional file 4 — Diet related policies meta-analysis with Rappaport, Daskalakis and Sendacki [66] replacing Foster, et al. [55]. [file 1479-5868-10-101-S4.docx]

**Additional file 4 -** Replacing Foster, *et al.* 2008 [55] with Rappaport, Daskalakis and Sendecki, 2013 [66]

**Other diet related policies** – random effects meta-analysis

|  |  |  | **Correlation between pre- and post- scores** | | | | | | | | | | | |
| --- | --- | --- | --- | --- | --- | --- | --- | --- | --- | --- | --- | --- | --- | --- |
|  |  |  | **r=0.4** | | | |  | **r=0.6** | | |  | **r=0.8** | | |
|  |  | **Study** | **ES** | **95% CI** | | **% Weight** |  | **ES** | **95% CI** | **% Weight** |  | **ES** | **95% CI** | **% Weight** |
| **Covariate outcome correlation or multiple correlation** | **r=0.1** | Fox, *et al.* 2009 [56] | -0.027 | -0.123, 0.069 | | 26.40 |  | -0.027 | -0.123, 0.069 | 26.40 |  | -0.027 | -0.123, 0.069 | 26.40 |
|  |  | Johnson, *et al.* 2012 [31] | -0.040 | -0.133, 0.053 | | 28.29 |  | -0.040 | -0.133, 0.053 | 28.29 |  | -0.040 | -0.133, 0.053 | 28.29 |
|  |  | Rappaport, Daskalakis and Sendecki, 2013 [66] | 0.005 | -0.102, 0.111 | | 21.48 |  | 0.005 | -0.102, 0.111 | 21.48 |  | 0.005 | -0.102, 0.111 | 21.48 |
|  |  | Veugelers and Fitzgerald, 2005 [58] | -0.026 | -0.127, 0.075 | | 23.83 |  | -0.026 | -0.127, 0.075 | 23.83 |  | -0.026 | -0.127, 0.075 | 23.82 |
|  |  | **D+L pooled ES** | -0.024 | -0.073, 0.026 | | 100.00 |  | -0.024 | -0.073, 0.026 | 100.00 |  | -0.024 | -0.073, 0.026 | 100.00 |
|  |  | **Heterogeneity** | I^2^ = 0.0%, p=0.941 | | |  |  | I^2^ = 0.0%, p=0.941 | |  |  | I^2^ = 0.0%, p=0.941 | |  |
|  |  |  |  | |  |  |  |  |  |  |  |  |  |  |
|  | **r=0.3** | Fox, *et al.* 2009 [56] | -0.026 | | -0.127, 0.075 | 25.24 |  | -0.026 | -0.127, 0.075 | 25.24 |  | -0.026 | -0.127, 0.075 | 25.24 |
|  |  | Johnson, *et al.* 2012 [31] | -0.038 | | -0.127, 0.051 | 32.24 |  | -0.038 | -0.127, 0.051 | 32.24 |  | -0.038 | -0.127, 0.051 | 32.24 |
|  |  | Rappaport, Daskalakis and Sendecki, 2013 [66] | 0.005 | | -0.106, 0.115 | 21.04 |  | 0.005 | -0.106, 0.115 | 21.04 |  | 0.005 | -0.106, 0.115 | 21.04 |
|  |  | Veugelers and Fitzgerald, 2005 [58] | -0.026 | | -0.135, 0.083 | 21.49 |  | -0.026 | -0.135, 0.083 | 21.49 |  | -0.026 | -0.135, 0.083 | 21.49 |
|  |  | **D+L pooled ES** | -0.024 | | -0.074, 0.027 | 100.00 |  | -0.024 | -0.074, 0.027 | 100.00 |  | -0.024 | -0.074, 0.027 | 100.00 |
|  |  | **Heterogeneity** | I^2^ = 0.0%, p=0.949 | | |  |  | I^2^ = 0.0%, p=0.949 | |  |  | I^2^ = 0.0%, p=0.949 | |  |
|  |  |  |  | |  |  |  |  |  |  |  |  |  |  |
|  | **r=0.5** | Fox, *et al.* 2009 [56] | -0.024 | | -0.127, 0.080 | 23.41 |  | -0.024 | -0.127, 0.080 | 23.41 |  | -0.024 | -0.127, 0.080 | 23.41 |
|  |  | Johnson, *et al.* 2012 [31] | -0.035 | | -0.116, 0.046 | 38.38 |  | -0.035 | -0.116, 0.046 | 38.38 |  | -0.035 | -0.116, 0.046 | 38.38 |
|  |  | Rappaport, Daskalakis and Sendecki, 2013 [66] | 0.004 | | -0.109, 0.117 | 19.71 |  | 0.004 | -0.109, 0.117 | 19.71 |  | 0.004 | -0.109, 0.117 | 19.71 |
|  |  | Veugelers and Fitzgerald, 2005 [58] | -0.026 | | -0.142, 0.090 | 18.51 |  | -0.026 | -0.142, 0.090 | 18.51 |  | -0.026 | -0.142, 0.090 | 18.51 |
|  |  | **D+L pooled ES** | -0.023 | | -0.073, 0.027 | 100.00 |  | -0.023 | -0.073, 0.027 | 100.00 |  | -0.023 | -0.073, 0.027 | 100.00 |
|  |  | **Heterogeneity** | I^2^ = 0.0%, p=0.959 | | |  |  | I^2^ = 0.0%, p=0.959 | |  |  | I^2^ = 0.0%, p=0.959 | |  |

95% CI; 95% confidence interval, ES; effect size, D+L; DerSimonian and Laird
